# Type IV minor pilin ComN predicted the USS-receptor in Pasteurellaceae

**DOI:** 10.3389/fmicb.2025.1647523

**Published:** 2025-10-31

**Authors:** Stian Aleksander Helsem, Kristian Alfsnes, Stephan A. Frye, Alexander Hesselberg Løvestad, Ole Herman Ambur

**Affiliations:** ^1^Department of Life Sciences and Health, Faculty of Health Sciences, OsloMet, Oslo, Norway; ^2^Division for Infection Control, Norwegian Institute of Public Health, Oslo, Norway; ^3^Division of Laboratory Medicine, Department of Microbiology, Oslo University Hospital, Oslo, Norway

**Keywords:** Uptake Signal Sequence, USS, prepilin peptidase-dependent protein A, PpdA, DNA-binding protein, PulG/HofG, competence protein N, ComN

## Abstract

The Uptake Signal Sequence (USS) receptor, which facilitates the acquisition of homologous DNA by natural transformation in *Haemophilus influenzae* and other members of the Pasteurellaceae, remains unknown. Through discriminating functional gene ontology assessment, cellular localization predictions, and deep-learning structural modeling of protein-DNA complexes, prepilin peptidase-dependent protein A (PpdA) was identified as the strongest USS receptor candidate in different Pasteurellaceae family members with divergent USS specificities. Pasteurellaceae PpdA (PpdA_Past_) was the only orthogroup modeled by AlphaFold3 (AF3) to form specific complexes with USS significantly better than complexes with sequence-scrambled versions of USS. Further analyses of PpdA-USS complexes using geometric deep learning protein-DNA sequence specificity predictions and coevolution analyses were found to further support PpdA as the USS receptor candidate in 10 different Pasteurellaceae enriched with divergent USS dialects. PpdA_Past_ was predicted to possess USS-binding specificity by DeepPBS and was found to strongly coevolve with USS relative to other orthogroups. PpdA_Past_ was found to share both structural features and functionally involved electropositive residues with other DNA-binding minor pilins and the largely unexplored *Escherichia coli/Enterobacteriaceae* PpdA ortholog. One robust DNA-binding mode was identified with two alternative and opposite USS orientations. Combining modeled PpdA-USS proximity and coevolved signals revealed how the C-terminal region of PpdA_Past_ fitted one of two 180° alternative USS orientations. Root Mean Square Deviations (RMSDs) from molecular dynamics simulations of PpdA_Past_ USS complexes found reliable structures under 3 Å in moderately stable trajectories. The *ppdA* gene, previously documented as essential for transformation and constituting part of the competence regulon in *Haemophilus influenzae* and *E. coli*, was found in a conserved genomic location with conserved operonic organization across the Pasteurellaceae. Together, the *in silico* results of this study and the documented knock out phenotype make a strong case for PpdA_Past_ as the USS-receptor and provide future directions for recombinant PpdA_Past_ assays and *in vivo* experiments with mutants. Here, we propose ComN for use with these PpdA_Past_ orthologs in compliance with the previously assigned gene name and the predicted central role in competence as a DNA receptor with USS specificity.

## Introduction

Deep-learning protein structure prediction has revolutionized biological research in recent years, an impact that earned the 2024 Nobel Prize in Chemistry. The development of new tools and benchmarking studies involving crystallographic detail has allowed the field to obtain better functional understanding from predicted 3D structures, although models cannot represent ground truths (Varadi et al., 2025). Recently, tools that model interactions between proteins and ligands, such as DNA/RNA, have been developed and applied to address specific biological phenomena. Three such tools, AlphaFold3 (AF3), RosettaFold2NA, and Chai-1, have been developed to model protein-DNA complexes (Abramson et al., 2024; Baek et al., 2024; Krishna et al., 2024). The input data consists of protein and DNA sequences, which are linear amino acid and nucleotide sequences, respectively, that are folded in learned statistical pattern recognition algorithms. In a previous study, these three AI tools were applied to study modeled complexes of DNA and the minor type IV pilin ComP, which is the DNA Uptake Sequence (DUS)-specific protein in the bacterial Neisseriaceae family (Helsem et al., 2025). Several deep-learning algorithms have recently been developed to address a general need to go from protein-DNA complex structure modeling to DNA-binding specificity prediction. The Deep Predictor of Binding Specificity (DeepPBS) takes an approach that combines geometric convolution predictions of protein and DNA structures (Mitra et al., 2024). DeepPBS input data can be either experimentally generated, simulated, or predicted structures. This approach was cross-validated and benchmarked for a range of protein families (Mitra et al., 2024).

Natural transformation is the uptake and homologous recombination of DNA from the extracellular environment, a feature of many bacterial species (Niu et al., 2025). In contrast to other horizontal gene transfer mechanisms, such as conjugation and transduction, evolved to proliferate through the introduction of novel DNA and traits to bacterial cells, competence for transformation is an evolved trait of the recipient cell. Transformation is limited by different molecular mechanisms that bias DNA uptake to involve homologous DNA, which finally concludes the transformation process by homologous recombination and allelic replacement (Ambur et al., 2016). Competence for transformation is also a tightly regulated physiological state in most characterized bacteria (Dubnau and Blokesch, 2019). A unifying feature of the DNA uptake step in the transformation process of most Gram-negative species involves type IV pili (T4P), which are used to capture extracellular DNA (Dubnau and Blokesch, 2019). Current models show how DNA binds to the protruding pilus and is pulled onto the plasma membrane together with the depolymerizing and retracting pilus. Passage of DNA through the plasma membrane occurs in a single-stranded form through the transmembrane ComEC pore, conserved in both Gram-positive and Gram-negative bacteria. Once in the cytoplasm, DNA is processed by DprA, and homologous recombination is facilitated by RecA, a ubiquitous protein found in both bacteria and eukaryotes involved in DNA repair and allelic reshuffling. Extracellular DNA-binding may be either specific or nonspecific. Neisseriaceae (ꞵ-proteobacteria) and Pasteurellaceae (γ-proteobacteria) are the only families known to discriminate homologous from heterologous DNA at this initial step in the transformation process by specific binding to short (ca. 9–12 nt) DNA motifs, which are highly enriched in respective genomes (Sisco and Smith, 1979; Deich and Smith, 1980; Danner et al., 1980; Goodman and Scocca, 1988; Elkins et al., 1991; Mell et al., 2012; Treangen et al., 2008). Notable human pathogens in these families are *Neisseria gonorrhoeae* and *Neisseria meningitidis* of the Neisseriaceae, *H. influenzae*, and *Pasteurella multocida* of the Pasteurellaceae. The physiological state of competence for transformation is constitutive in *Neisseria*, whereas it is induced by cyclic AMP (cAMP) in a competence activator Sxy-dependent manner in *H. influenzae* and other bacteria (Dorocicz et al., 1993; Chandler, 1992). The induction of expression of 17 Sxy-dependent cyclic AMP receptor protein site-regulated (CRP-S) genes in *H. influenzae* is required for transformation (Sinha et al., 2020). The CRP-S regulon in *H. influenzae* encompasses 26 genes, many of which have well-characterized functions in the transformation process from DNA uptake to recombination. In the CRP-S regulon, the *comNOPQ* operon encoding four proteins of unknown functions, unknown cellular location(s), and without identified homologs, is required for DNA uptake and transformation in *H. influenzae* using in-frame gene deletions (Sinha et al., 2020). A prepilin peptidase signal was annotated to ComN and signal peptidase I signals in the ComOPQ proteins (Sinha et al., 2020).

The short DNA motifs recognized by Pasteurellaceae and Neisseriaceae are named Uptake Signal Sequence (USS) and DNA Uptake Sequences (DUS), respectively. USS and DUS are highly dissimilar in sequence motif and are therefore considered to have evolved independently and convergently to achieve the same means, i.e., to bias transformation to involve homologous DNA (Mell et al., 2012). Characteristic USS/DUS containing genomes harbor hundreds or thousands of these motifs, which are often organized as inverted repeats with secondary functions to DNA uptake as rho-independent transcriptional terminators, yet the presence of a single motif is sufficient to increase transformation rates (Mell et al., 2012; Ambur et al., 2007). The DUS and USS motifs have both evolved into distinct dialects/variants or types within each bacterial family and may differ by a few nucleotides from each other, yet with their respective transformation and uptake-essential inner cores conserved (Frye et al., 2013; Mell et al., 2012). The inner consistent core sequences are 5′-CTG-3′ in the Neisseriaceae DUS and 5′-GCGG-3′ in the Pasteurellaceae USS (Frye et al., 2013; Mell et al., 2012). A total of eight dialects have been described in the Neisseriaceae (AT-DUS, AG-DUS, AG-mucDUS, AG-eikDUS, AG-kingDUS, AA-king3 DUS, TG-wadDUS, and AG-simDUS) and two USS-types in the Pasteurellaceae classified as *Haemophilus*-type *Hin-*USS (5′-AAGT**GCGG**T-3′) and *Actinobacillus pleuropneumoniae*-type *Apl-*USS (5′-ACAA**GCGG**T-3′) (dialect differences underlined and USS inner core in bold) (Frye et al., 2013; Mell et al., 2012). Both DUS and USS dialects adhere to whole-genome phylogenies with their respective genomic enrichments within defined subclades (Frye et al., 2013; Mell et al., 2012; Redfield et al., 2006). DUS are confined to continuous motifs extending up to about 13 nucleotides in length, whereas the USS are discontinuous and are characterized by the 9 nt cores and two less conserved and discontinuous downstream AT-rich regions, making the USS motifs extend up to 32 nucleotides (Redfield et al., 2006). These AT-rich regions, spaced at approximately two full helical turns downstream of USS, are anticipated to contribute to DNA melting and facilitate kinking of the DNA helix in the USS inner core sequence to accommodate DNA entry through the outer membrane pore (Redfield et al., 2006).

The DUS-binding protein is the type IV minor pilin ComP (Cehovin et al., 2013; Berry et al., 2016; Berry et al., 2019), whereas the USS-specific protein remains unidentified. Since the components of the type IV pilus (T4P) machinery are conserved between Neisseriaceae and the Pasteurellaceae, it has been proposed that candidates for USS-specificity may be found at the T4P tip or in the many surface-exposed proteins (Mell et al., 2012). Only one targeted search for the USS-receptor has previously been undertaken, which excluded ComE1 as a candidate in *H. influenzae* (Molnar, 2004). Genome-wide sequencing maps of DNA uptake in *H. influenzae* demonstrated that USS biases the DNA uptake step, which is initiated by extracellular DNA-binding (Mora et al., 2020). Support for the general involvement of minor type IV pilins in transformation (specific and nonspecific) was found in the γ-Proteobacterium *Legionella pneumophila* (Braus et al., 2022). Here, the minor type IV pilin FimT was shown to be important for DNA uptake in *L. pneumophila*, as well as for DNA-binding in *Pseudomonas aeruginosa* and *Xanthomonas campestris* (Braus et al., 2022). FimT orthologs were also identified across a range of γ-proteobacteria, and particularly common FimT representatives were additionally identified in Xanthomonadales, Alteromonadales, and Pseudomonadales (Braus et al., 2022). Like ComP (Cehovin et al., 2013), the 3D structure of FimT displays an electropositive stripe, which was found to be associated with DNA-binding in electromobility shift (EMSA) and chemical shift perturbation (CSP) assays (Braus et al., 2022). The existence of a DNA-binding receptor on the *H. influenzae* surface was anticipated more than half a century ago (Scocca et al., 1974). This study aimed to comprehensively explore USS-receptor candidates using a structural modeling approach involving the latest deep-learning tools.

## Materials and methods

**Figure 1:**
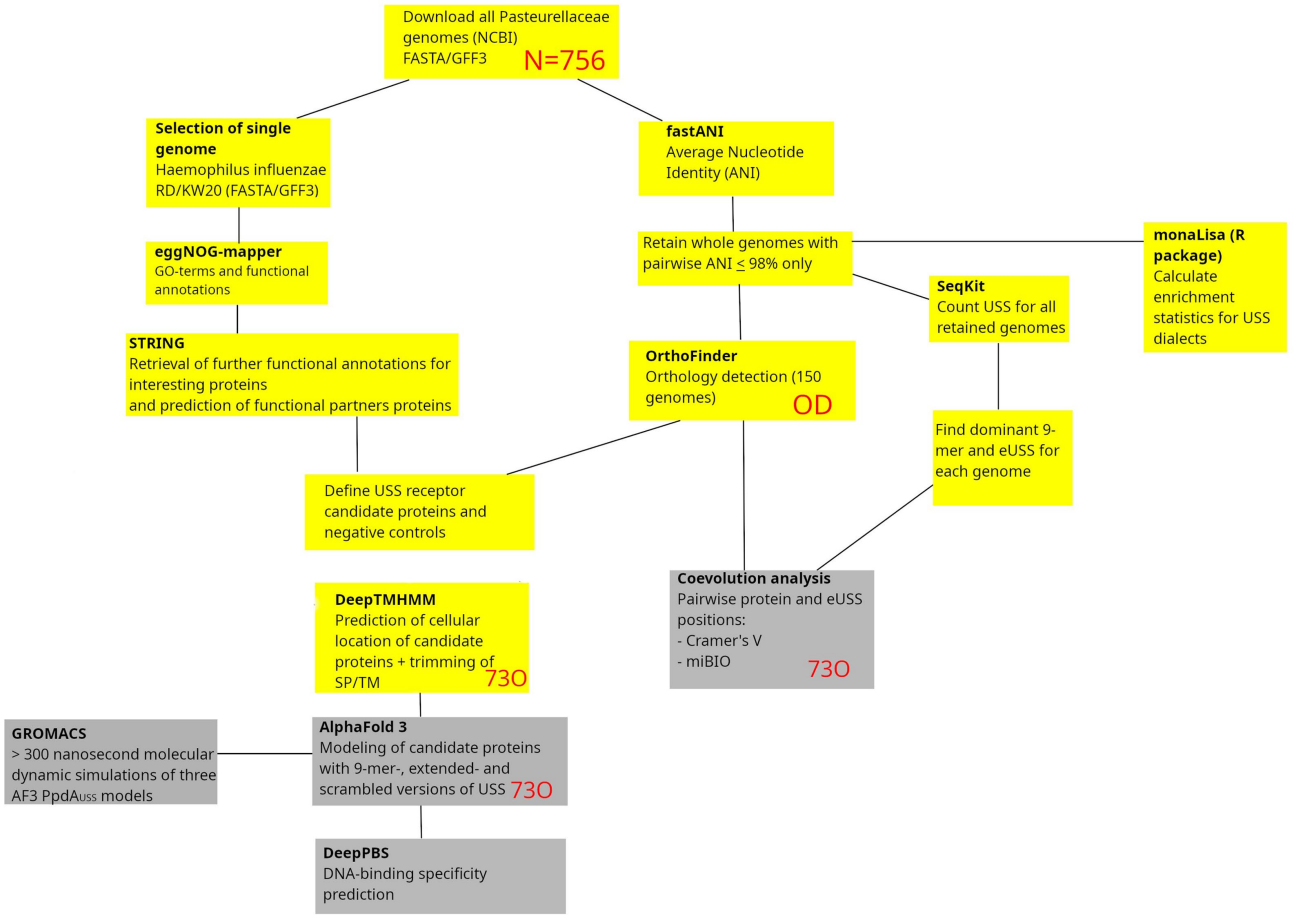
Methodological pipeline in the study. Starting with the download of all Pasteurellaceae genomes, the study takes two connected routes, one leading to coevolution analysis and the other to protein-DNA structural modeling and DNA-binding specificity predictions. Outputs from the yellow boxes are preparatory outputs reported in the Supporting Information, whereas the gray boxes are results reported in the Results section. SP, Signal peptides; TM, Transmembrane helices. Red letters refer to analysis output/input.

The search for USS-receptor candidates was initiated by downloading all available Pasteurellaceae genomes from NCBI in FASTA/GFF3 format, as outlined in Figure 1. A list of confidence metrics in protein and protein-DNA modeling, along with the corresponding abbreviations used, is listed in Table 1.

**Table 1:** Confidence metrics in protein and protein-DNA modeling and abbreviations.

Metrics and abbreviations	Explanations
ipTM Predicted interface template modeling score	Confidence range 0–1, where the range >0.6 was used here to determine potential DNA-binding properties of different proteins/orthogroups. Significant differences between PpdA_USS_ and PpdA_Scr_ are also reported.
PAE Predicted aligned error	Continuous variable with unit Ångstroms (Å); lower values indicate less error and higher confidence in structure. Significant differences between PpdA_USS_ and PpdA_Scr_ are reported.
pLDDT Predicted local distance difference test	Continuous variable with range 0–100, disulfide bridge where values >70 generally are considered confident (protein backbone usually correct, local errors possible). Significant differences between PpdA_USS_ and PpdA_Scr_ are reported.
CPPM Chain pair PAE minimum	A measure used to distinguish probable DNA binders from non-binders in interactions between protein and DNA chains. Significant differences between PpdA_USS_ and PpdA_Scr_ are reported.
DockQ	Continuous quality measure of similarity with confidence range 0–1 for docking models contained in two different PDB/mmCIF files.
RMSD Root mean square deviation of atomic positions	A structural difference measure in Å units used here to compare structural differences over time relative to input structure in GROMACS.
1-RMSD One minus RMSD	A structural similarity measure in Å units used here to compare structural similarities of PpdA_USS_ complexes over time in GROMACS relative to input structure.
AF and AF3 AlphaFold and AlphaFold3	Protein structure modeling algorithm and Protein-ligand (here DNA) structure modeling algorithm
OD OrthoFinder dataset	Comprising 293 orthogroups listed in Supplementary Table S2, identified by Orthofinder in 756 Pasteurellaceae genomes
73O 73 orthogroups	Part of the OD modeled in AF3 comprises 73 orthogroups
Φ*c* Cramér’s Phi	Range 0–1. A measure of association between two categorical variables, which here were single pairwise positions in protein and eUSS alignments used in co-evolution assessment
Φ*_C_* factor Cramérs Phi factor	Continuous variable denoting the total number of significant Φ*_C_* values divided by the length of the orthogroup sequence
MI Mutual information	Continuous variable in miBIO scoring output with values for pairwise position associations used here across orthogroup alignments and eUSS
MI factor Mutual information factor	Number of positive MI values divided by the length of the sequences in the orthogroup
eUSS Extended USS	The two extended USS dialects with 9-mer core USS in bold and dialect differences underlined: e*Hin*-USS 5′-A**AAGTGCGGT**CAATTTT-3′ e*Apl*-USS 5′-A**ACAAGCGGT**CAAATTT-3′

### eggNOG-mapper and STRING - discriminating unlikely USS receptor candidate proteins

eggNOG-mapper v2.1.12 (Cantalapiedra et al., 2021; Huerta-Cepas et al., 2019) was used to assign all proteins in the *H. influenzae* Rd. proteome to orthologous groups in the eggNOG database (Figure 1). Diamond v. 2.1.10 (Buchfink et al., 2021) was employed as the sequence search aligner, set to ultra-sensitive mode to maximize homolog retention. The option to annotate with experimental and non-electronic (--all) Gene Ontology (GO) evidence was used to ensure comprehensive functional classification. To save computational time and optimize the accuracy of annotations, the parameters tax_scope and tax_scope_mode were set to Gammaproteobacteria and Bacteria, respectively. The output was then screened and filtered, applying GO terms and functional descriptions considered relevant to USS receptor function, listed in Supplementary Table S1.

This annotated eggNOG output contained functional annotations for 296 *H. influenzae* Rd. proteins, which were then queried against the STRING database using an in-house script^1^ to retrieve further functional annotations and top-ranked interaction partners (Supplementary Table S2).

### fastANI and OrthoFinder

To identify orthologs of USS receptor candidate proteins, fastANI v. 1.34 (Jain et al., 2018) was used to calculate the average nucleotide identity (ANI) across all available (*N* = 756) *Pasteurellaceae* genomes as of December 4, 2024, using the many-to-many approach as per instructions in the fastANI GitHub repo.^2^ Using an in-house script, genomes were clustered together based on a minimum reciprocal ANI of 98%, and for each cluster, one representative genome for each species was retained, with 150 genomes remaining for downstream analysis. OrthoFinder v. 3.0.1b1 (Emms and Kelly, 2019) was then used to perform orthology detection for this dataset using Diamond v. 2.1.10 (Buchfink et al., 2021) as the sequence search engine and the option to infer gene trees from MAFFT v. 7.525 (Katoh, 2002; Katoh and Standley, 2013) sequence alignments. The resulting data was referred to as the OrthoFinder dataset (OD), comprising 293 orthogroups. Accession numbers for the OD are listed in Supplementary Table S2. USS receptor candidacy was then assessed and filtered based on (i) functional relevance in eggNOG and STRING annotations and (ii) orthogroup coverage (number of OD genomes with ortholog present) of at least 90% in the OD. After applying these filters, the final number of USS receptor candidates was reduced to 73 proteins (73O), as highlighted in Supplementary Table S2, while 218 of the OD proteins were deemed unlikely to be the USS receptor.

### DeepTMHMM, AlphaFold3, and DeepPBS

DeepTMHMM is a deep-learning protein language model-based tool that predicts the topology of transmembrane proteins (Hallgren et al., 2022) and provides information that can educate the understanding of the cellular localization of proteins. An earlier version of DeepTMHMM (TMHMM v. 2.0) (Sonnhammer et al., 1998; Krogh et al., 2001) was also previously used to focus the USS-receptor search for cell surface-exposed proteins (Molnar, 2004). DeepTMHMM v. 1.0.24 was used here to predict the cellular localization of orthologs from 10 selected species represented in the OD for the USS candidates listed in Supplementary Table S2, each highly enriched in *Hin-*USS or *Apl-*USS. The 10 species were *Hin*-USS enriched species *Haemophilus influenzae* Rd., *Aggregatibacter* sp. oral taxon 513, *Aggregatibacter actinomycetemcomitans* strain 31S, *Pasteurella multocida* strain NCTC8282, *Pasteurellaceae bacterium* Orientalotternb1, and *Apl*-USS enriched species *Mannheimia succiniciproducens* strain MBEL55E, *Mannheimia bovis* strain 39324S-11, *Actinobacillus equuli* subsp. haemolyticus strain 3524, *Actinobacillus lignieresii* strain NCTC4189, and *Frederiksenia canicola* strain HPA 2 (highlighted in Supplementary Table S2 with USS-counts). Since USS-binding takes place before passage of the outer membrane into a DNase-protected stage (Mora et al., 2020) and current natural transformation models show extracellular DNA-binding (Dubnau and Blokesch, 2019). We were interested in domains predicted to face the exterior environment. The DeepTMHMM predictions were thus used to guide modeling of orthologous protein domains with DNA in AF3. When DeepTMHMM predicted a single extracellular domain for an ortholog, this domain was selected for AF3 modeling. In cases where an ortholog was predicted with multiple extracellular domains, or the extracellular domain in single-domain proteins was shorter than 33 residues, well below the limit of known functional domains (Jones et al., 1998). The full-length protein sequences were used for AF3 modeling.

The 73 orthologous proteins and domains (73O) were then modeled in AF3 together with the species’ dominant 9-mer USS dialect (orthogroup_USS_) and scrambled forms of the USS dialect (orthogroup_scr_), each running a minimum of 20 replicates with different seeds. The reasons for doing this were two-fold: 1. To discriminate on modeling robustness, expecting higher confidence metrics for the true USS receptor relative to other orthogroups. 2. To discriminate on modeled USS-binding specificity, expecting statistically significantly higher confidence metrics for orthogroup_USS_ predictions than orthogroup_scr_ predictions for the true USS receptor compared to other orthogroups. For the AF3 output models, an interface predicted Template Modeling (ipTM) range of 0.6 < = 1 (ipTM_r_) was used to determine potential DNA-binding properties of proteins. Furthermore, we ran ipTM, Predicted Aligned Error (PAE), Chain Pair PAE minimum (CPPM), and the predicted Local Distance Difference Test (pLDDT). Wilcoxon rank sum tests on orthogroup_USS_ vs. orthogroup_scr_ results for predictions within ipTM_r_ as previously described (Helsem et al., 2025). Based on the strength of interactions between the proteins and the DNA as measured by the lower cut-off ipTM value of 0.6 (Section 3: Interpreting results from AlphaFold server, n.d.), as well as a lower predicted aligned error (PAE) for orthogroup_USS_ compared to orthogroup_scr_, we identified the strongest USS-specific binding orthogroup candidate to be prepilin peptidase dependent protein A (PpdA). Different names assigned to PpdA in *H. influenzae* Rd. were Uncharacterized protein HI0938 (Swiss-Prot), pilus assembly FimT family protein (RefSeq), prepilin-type cleavage/methylation domain-containing protein (RefSeq), type II secretion system protein (INSDC), type II secretion system GspH family protein (INSDC), PulG (VanWagoner et al., 2004), and Tfp pilus assembly protein FimT/FimU (INSDC). The *H. influenzae* Rd. gene HI0938 had previously been designated as *comN* (Molnar, 2004).

DockQ (Mirabello and Wallner, 2024), a measure of similarity quality, as calculated for pairwise orthogroup_USS_ top models (ipTM) for all 10 modeled Pasteurellaceae species. Previously, a graph-edge method was used to place models in clusters where all members have reciprocal DockQ values > = 0.490. Using 0.490 as a lower DockQ threshold instead of 0.8 was a good compromise for cluster size and number, which we wanted to increase and decrease, respectively. No models were permitted to be allocated in multiple clusters. For each species, the models were superimposed on the largest clusters to the α-carbons and visualized in overlays using PyMOL v. 3.0.0 (Mitra et al., 2024) Open Source (Molecular Graphics System, Schrödinger, LLC).

We explored whether the geometric deep-learning algorithm, DeepPBS v. 1.0 (Mitra et al., 2024), could predict the DNA-binding specificity of the AF3-predicted USS receptor to match the USS motif across distinct Pasteurellaceae family orthologs. From a protein structure, DeepPBS aggregates the atom environment (type, charge, radius), which is laid upon a symmetrized DNA helix structure (sym-helix) to which a bipartite geometric convolution is made. This overall convolution is informed by DNA groove and DNA shape readouts consisting of major and minor groove convolutions (groove readout, i.e., not base readout) and DNA backbone sugar and phosphate convolutions (shape readout). We ran DeepPBS using the default module, which considers shape and groove properties of the DNA (“both readout”) without introducing any DNA-sequence bias. We reasoned that if this approach predicted binding specificity to match the two different USS sequences (*Hin-*USS and *Apl-*USS), it would provide additional support for the AF3 modeling. Assuming that the DNA-binding specificity of the USS-receptor would be reflected as an evolutionary trace in the genomic conservation of each nucleotide in the USS, DeepPBS predictions and sequence logos of genomic USS conservation were compared. The sequence logos were generated as devised by Redfield et al. (2006), allowing one mismatch to the 9-mer USS. Finding correlations between USS conservation and DeepPBS predicted specificity could further establish a functional connection between predicted specificity and the existing genomic USS. Finally, in a series of negative control experiments, we explored whether DeepPBS could predict any DNA-position relative nucleotide preference *de novo* using modeled PpdA_Scr_ to suggest nucleotide biases.

DeepPBS outputs probability distributions of the four nucleotides in each position of one DNA strand (Watson) of the input protein-DNA complex, heavy-atom relative importance scores for the protein, and a nucleotide binding-specificity plot for an input protein-DNA complex. The mean predicted nucleotide probabilities at each USS position from 10 USS-receptor candidates were calculated separately, and the outputs were combined according to USS-dialect (*Hin-*USS and *Apl-*USS). One-sample t-tests were carried out to check for mean predicted nucleotide probabilities significantly deviating from 0.25 (random chance for any nucleotide) at each USS position. Ensembles of DeepPBS nucleotide binding-specificity plots were animated by first converting SVG to PNG using Inkscape v. 1.4-dev (Inkscape Project, version 1.4)^3^ and then concatenating PNGs to GIF format using ImageMagick v. 6.9.12–98 (ImageMagick ISLL) (Supplementary Videos S1, S2).

### USS statistics, extended USS, and coevolution analysis

To inform the structural modeling, prediction of sequence-specificity, and coevolution analyses, the per-genome numbers of *Hin-*USS and *Apl-*USS of all genomes represented in OD were extracted using SeqKit v. 2.3.0 (Shen et al., 2016) and corrected for genome size (USS counts/Mb) (Supplementary Table S3). For structural modeling and coevolution analysis specifically, we widened the 9-mer USS to the 17-mer extended USS (eUSS), extending the USS one position upstream and seven positions downstream into less conserved, yet potentially evolutionarily informative regions (Table 1). Individual nucleotide positions in the eUSS were numbered 1–17. The genome-specific eUSS were calculated from an alignment of all genomic occurrences of the dominant 9-mer USS (*Hin-*USS/*Apl-*USS). Regarding specific orthogroup-eUSS pairwise positions, only the eUSS positions in the alignment that were variable had the potential to detect coevolved pairs in the coevolution analyses (see below). eUSS positions 2, 5–9, and 15–16 are invariant in the two USS dialects and hence not informative.

Pasteurellaceae genomes statistically overrepresented in either USS dialect were considered informative for the orthogroup-USS coevolution analysis. Thus, enrichment statistics for the USS dialects were calculated for all 150 source genomes for the OD (Supplementary Table S3). A Markov model of order 4 was used, following the recommendations in Davidsen (2004), although we used 9-mers instead of 10-mers. All 150 source genomes for OD were found to show an overrepresentation of either *Hin-*USS or *Apl-*USS to variable extents (from 31.34/Mb to 913.03/Mb, as shown in Supplementary Table S3) and were included in the coevolution analysis. Multiple sequence alignments (MSA) for the 73 AF3 modeled orthogroups (73O) were compared to MSAs of the calculated eUSS (Watson strand) systematically, using two different approaches as follows.

Cramér’s Φ (Φ*_C_*) was calculated for pairwise sites across orthogroup alignments and eUSS. Bonferroni correction of *p*-values was applied to account for multiple testing. Only the source genomes for OD having orthologs in each respective orthogroup were included in the calculation of Φ*_C_*. For orthogroups containing multiple copies for a species (paralogs), underscores were appended to the genome accession in both the orthogroup and eUSS MSAs to distinguish paralog 1, 2, etc. Φ*_C_* was calculated for all pairwise positions in the two alignments as a measure of coevolution between the orthogroups and eUSS. Values ranged from 0 for no correlation to 1 for perfect correlation. All correlations between eUSS and PpdA were recorded, and the three USS-dialect-specific positions (3–5 of eUSS) were traced specifically.

miBio (Camenares, 2020)^4^ was employed to calculate mutual information (MI) values across pairwise positions across orthogroup alignments (MSAs) and eUSS. The option to shuffle the identities of the residues and subtract shuffled MI values from the original ones was applied. As miBio allows for customization of grouping of residues, we used a custom grouping pairing amino acids D/E, K/R, and N/Q, because these have been shown to have comparable nucleotide-binding preferences in protein-DNA complexes (Luscombe, 2001; Hossain et al., 2024), as well as treating gaps as separate, valid states. Finally, the mean MI was calculated for the resulting positive MI values of each orthogroup. All MI correlations between eUSS and PpdA were recorded, and the three USS-dialect-specific positions (3–5 of eUSS) were compared to the corresponding Φ*c* values.

### Assessment of PpdA-(e)USS binding modes

To further investigate binding modes of PpdA_(e)USS_ (both PpdA_USS_ and PpdA_eUSS_), we used AF3 to model (e)USS in complex with PpdA from the rest of the 150 genomes represented in OD, having at least 300 USS per Mb genome size, to avoid giving weight to PpdAs from species that may have lost USS-specificity or impose very weak USS DNA-binding biases (Supplementary Table S3). At this point, since we had insight into the structure and pilin-associated function of PpdA, we N-terminally trimmed their sequences, as in the study of recombinantly expressed ComP (Berry et al., 2016), aligned on the DeepTMHMM prediction for *H. influenzae* Rd., as this also saved computational time. Subsequently, we clustered the top-ranking models based on a maximum l-RMSD <10, using the graph-edge clustering method previously used for DockQ, mentioned above. We omitted the use of DockQ here and instead used l-RMSD, since the term “fraction of native/common contacts” used as part of the DockQ measure is meaningless across homologous proteins/DNAs with differing position-wise residues.

### Molecular dynamics simulation—GROMACS

Three of the models from the binding mode assessment step were used as input in GROMACS (Groningen Machine for Chemical Simulations) v. 2025.0, which includes CUDA-GPU support (Páll et al., 2015; Abraham et al., 2015) for molecular dynamics simulation (MD), to study the stability and development of the respective binding modes. All molecular dynamics simulations were performed using the same parameter values, running for a minimum of 300 ns in a rhombic dodecahedron water box, using the AMBER ff14SB + parmbsc1 (Ivani et al., 2016) force field, the tip3p water model for solvent, and adding necessary ions to neutralize the charge of the protein-DNA complexes, using a NaCl concentration of 0.15 M. The temperature and reference pressure were set to 300 K and 1 bar, respectively. The simulation trajectories were visually inspected using the IPython widget NGLview (Nguyen et al., 2018), and all frames (default interval of 5 ps) of the last ns of the MD simulation were concatenated into a single PDB file. Furthermore, we used GROMACS’ in-built RMSD calculation to assess the structural similarity between all frames in the MD simulation relative to the input structure.

### Electrostatic maps

We used the Adaptive Poisson-Boltzmann Solver (APBS) (Baker et al., 2024) to calculate electrostatic maps for the PpdAs of the top-ranking AF3 model from the largest PpdAUSS cluster and the top-ranking models from the two largest clusters for PpdA_eUSS_, and to visualize the maps in PyMOL v. 3.0.0 (Molecular Graphics System, Schrödinger, LLC). This was also done for the last GROMACS frames for each of these three cases. Additionally, we attach the APBS-generated electrostatic maps for *Escherichia coli* PpdA (PpdA*_Ecol_*) and *L. pneumophila* FimT (FimT*_Lpne_*) (DNA not shown). For FimT*_Lpne_*, we observed large differences between the local and AF3 web server^5^ outputs and were able to reconcile these differences by locally running the jackhmmer, using v. 3.4 (Potter et al., 2018) and MSA search on the BFD database (Steinegger et al., 2019; Steinegger and Söding, 2018) using the parameter incdomE 0.01.

## Results

The search for the USS-receptor proceeded through a series of initial steps as described in the Materials and Methods section, and the outputs are reported in the Supporting Data (Supplementary Tables S1–S3), which involved functional gene ontology assessment, cellular location predictions, USS-enrichment statistics, and orthogroup coverage across USS-enriched genomes/species. These outputs were used in consecutive deep-learning structural modeling of protein-DNA complexes (refer to Supplementary Table S2 for all orthogroups modeled in AF3), specificities of DNA-binding predictions, and coevolutionary analysis described in each section below.

### Assessment of protein-DNA complexes modeled in AF3

**Figure 2:**
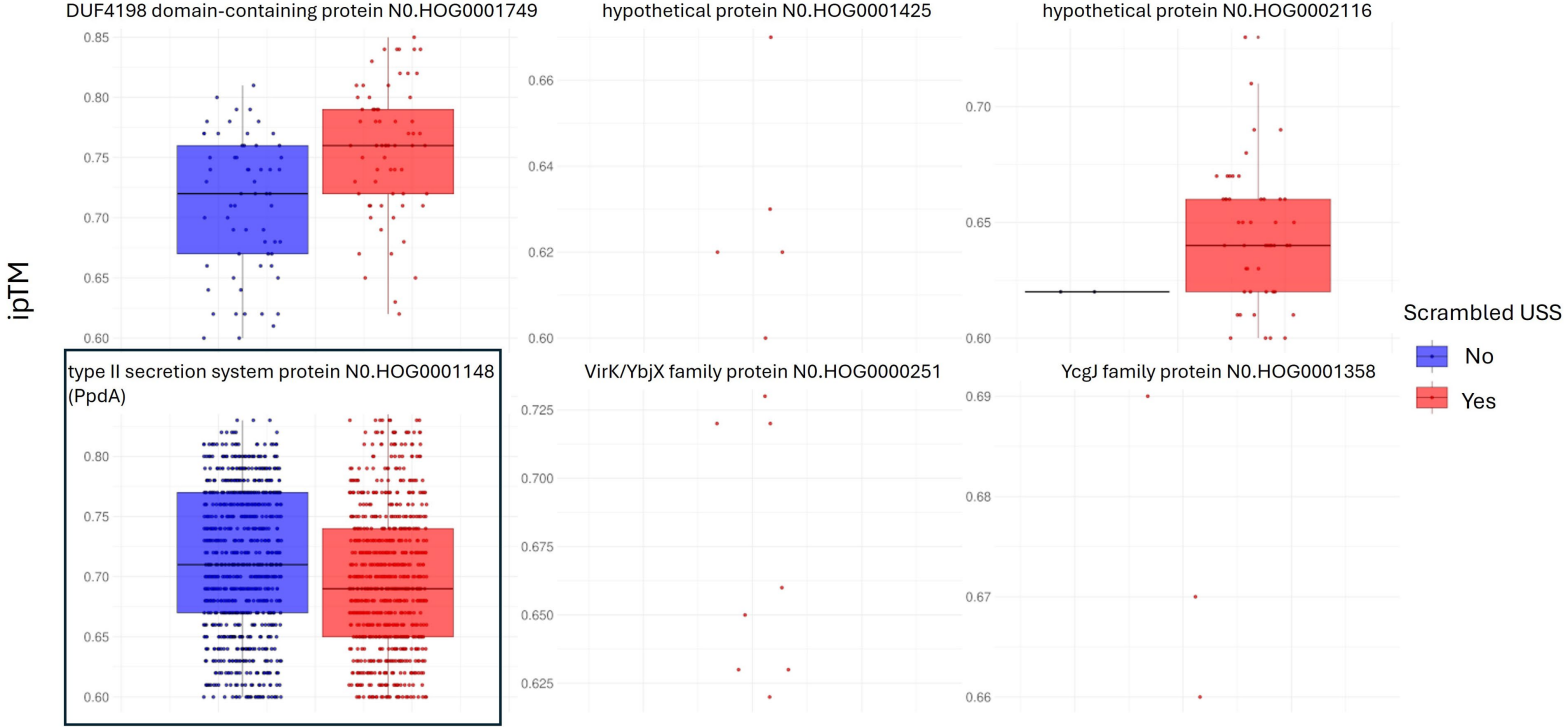
Distribution of model confidence ipTM values. All AF3 orthogroup_USS_ and orthogroup_scr_ models for the six orthogroups with results within ipTM_r_ are represented as box plots. 1,690 out of 2000 PpdA-DNA models (type II secretion system protein) in boxed frame, were within ipTM range and complexes with USS-DNA, PpdA_USS_, were the only group of protein-DNA complexes significantly of higher confidence (higher ipTM) relative to PpdA_scr_ complexes with scrambled DNA.

Out of 73 modeled orthogroups, AF3 modeled only six potential candidate DNA-binding orthogroups across all 10 species, with at least one model within ipTM_r_. These were PpdA, DUF4198 domain-containing protein, two hypothetical proteins, protein annotated “MULTISPECIES: hypothetical protein,” VirK/YbjX family protein, and YchJ family protein (Table 2). Figure 2 shows the ipTM distribution of all AF3 models generated within ipTM_r_ for these six proteins. A total of 9,400 models were built for the six orthogroups together, each modeled with either USS (orthogroup_USS_) or scrambled versions of USS (orthogroup_scr_). The rationale for comparing the USS and scrambled models was the expectation that AF3 would generate better quality models with USS than scrambled USS for the USS receptor. The statistical tests are reported in Code Output S1. The distributions of the three other modeling quality parameters, pLDDT, PAE, and CPPM, are plotted in Supplementary Figures S1–S3. Since all 10 modeled species had USS-enriched genomes and therefore were expected to have the USS receptor, the OrthoFinder orthogroup coverage was recorded for all six orthogroups in the 150 OD genomes (Supplementary Table S2), as shown in Table 2.

**Table 2:** AF3 modeling results for the six orthogroups with models within ipTMr.

Orthogroup	Models							
	OD 150 coverage	AF3 modeled prot-DNA complexes	Models in ipTMr	Modeled species (max. 10) w/complexes in ipTMr	ipTM USS vs. scr	pLDDT USS vs. scr	PAE USS vs. scr	CPPM USS vs. scr
PpdA	147	2,000	1,629	10	*** higher	*** higher	–	*** lower
Hyp. prot. N0.HOG0002116	44	400	51	2	–	*** lower	*** higher	–
DUF4198 domain-prot.	77	1,600	128	2	*** lower	*** lower	*** higher	*** higher
Hyp. prot. N0.HOG0001425	120	1,800	5	2	All scr	All scr	All scr	All scr
VirK/YbjX family protein	142	1,600	8	4	All scr	All scr	All scr	All scr
YchJ family protein	140	2,000	3	2	All scr	All scr	All scr	All scr

Numbers refer to orthogroup coverage in the OD of 150 genomes, number of AF3 models outside and inside ipTMr and the species coverage of models within ipTMr in the 10 modeled species enriched in either *Hin*- or *Apl*-USS. – signify non-significant differences between USS and scr models, and *** signify significant differences with *p* < 0.0001. Better models have higher ipTM and pLDDT and lower PAE and CPPM.

### PpdA_USS_ DeepPBS DNA-binding specificity predictions

**Figure 3:**
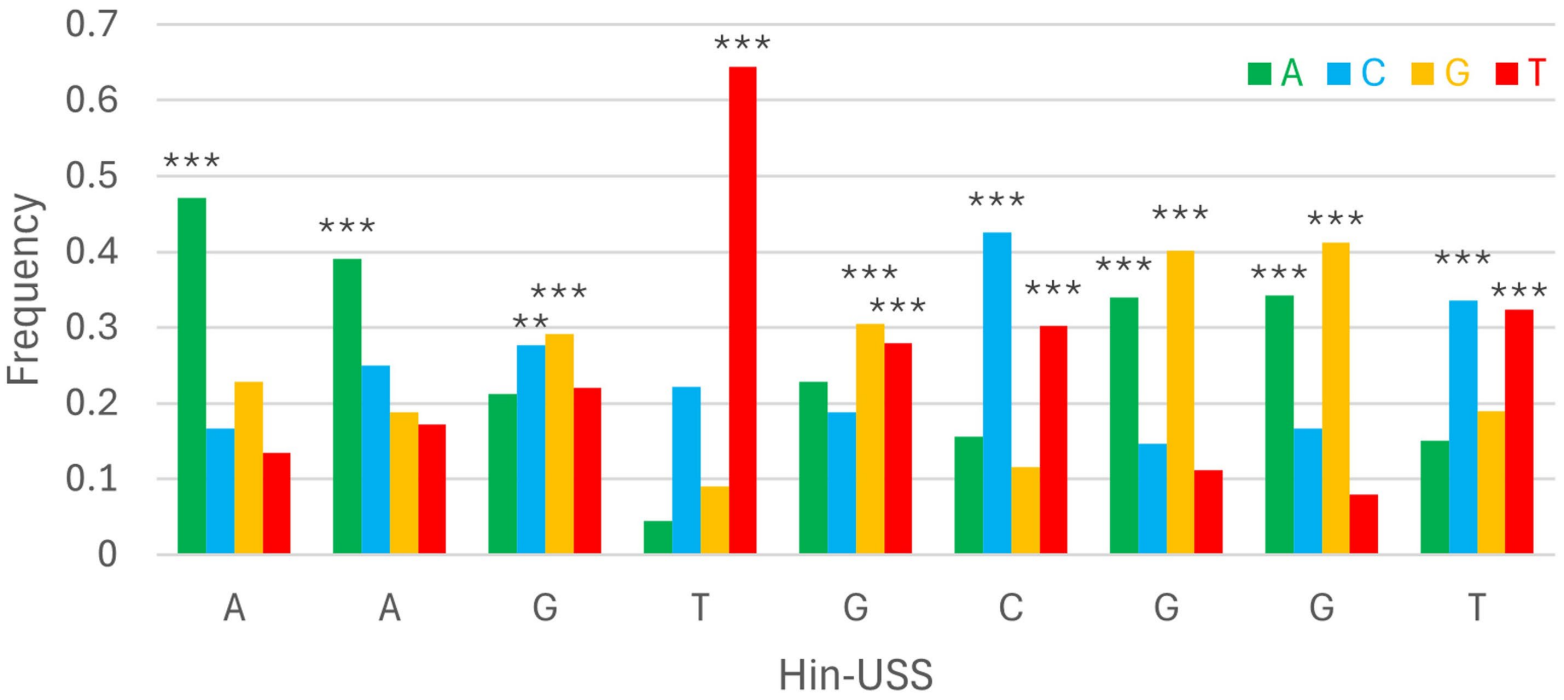
Consensus DeepPBS predictions of PpdA specificity against the reference *Hin-*USS. Distribution of consensus predicted nucleotide probabilities in each of the nine *Hin-*USS positions. Asterisks denote significant representation above random (0.25), ***p < 0.001, **p < 0.01, *p < 0.05.

DeepPBS was run on 808 PpdA_USS_ and 797 PpdA_scr_ AF3 models, which were within ipTM_r_. We present the DeepPBS results using the default module (“both readout”). The DeepPBS results for each PpdA from each of the 10 representative *Hin*-USS and *Apl*-USS species are detailed in Supplementary Figures S4, S5 and Supplementary Tables S4, S5. The average DeepPBS predictions of nucleotide representation from all five *Hin-*USS species revealed overrepresented nucleotides matching *Hin-*USS were found with strong significance (***) above random (25%) in all nine *Hin*-USS positions (Figure 3; Supplementary Table S4; Supplementary Video S1). The nucleotide probabilities for each USS position ranged from the highest 0.6448 for USS position 4(T) to the lowest 0.2917 for 3(G). Positions 1(A), 2(A), and 4(T) were predicted without any significant nucleotide ambiguities, whereas all other positions were predicted together with one other significant ambiguity: transversion permutations in positions 3(G/C) and 5(G/T) and transition permutations in positions 6(C/T), 7(G/A), 8(G/A), and 9(T/C). In descending order, the highest levels of overrepresentation were found in positions 4(T), 1(A), 6(C), 8(G), 7(G), 2(A), 9(T), 5(G), and 3(G). Furthermore, DeepPBS predicted the near-exact overrepresented nucleotides matching *Apl-*USS with variable significance (Supplementary Figure S6; Supplementary Table S4; Supplementary Video S2). DeepPBS runs with either the *Hin-*USS orthogroup_scr_ or *Apl-*USS orthogroup_scr_ models showed that all predictions failed to predict *de novo* a coherent signal different from random (Supplementary Table S5).

### Orthogroup-eUSS coevolution analyses by Cramér’s Φ and miBIO

**Figure 4:**
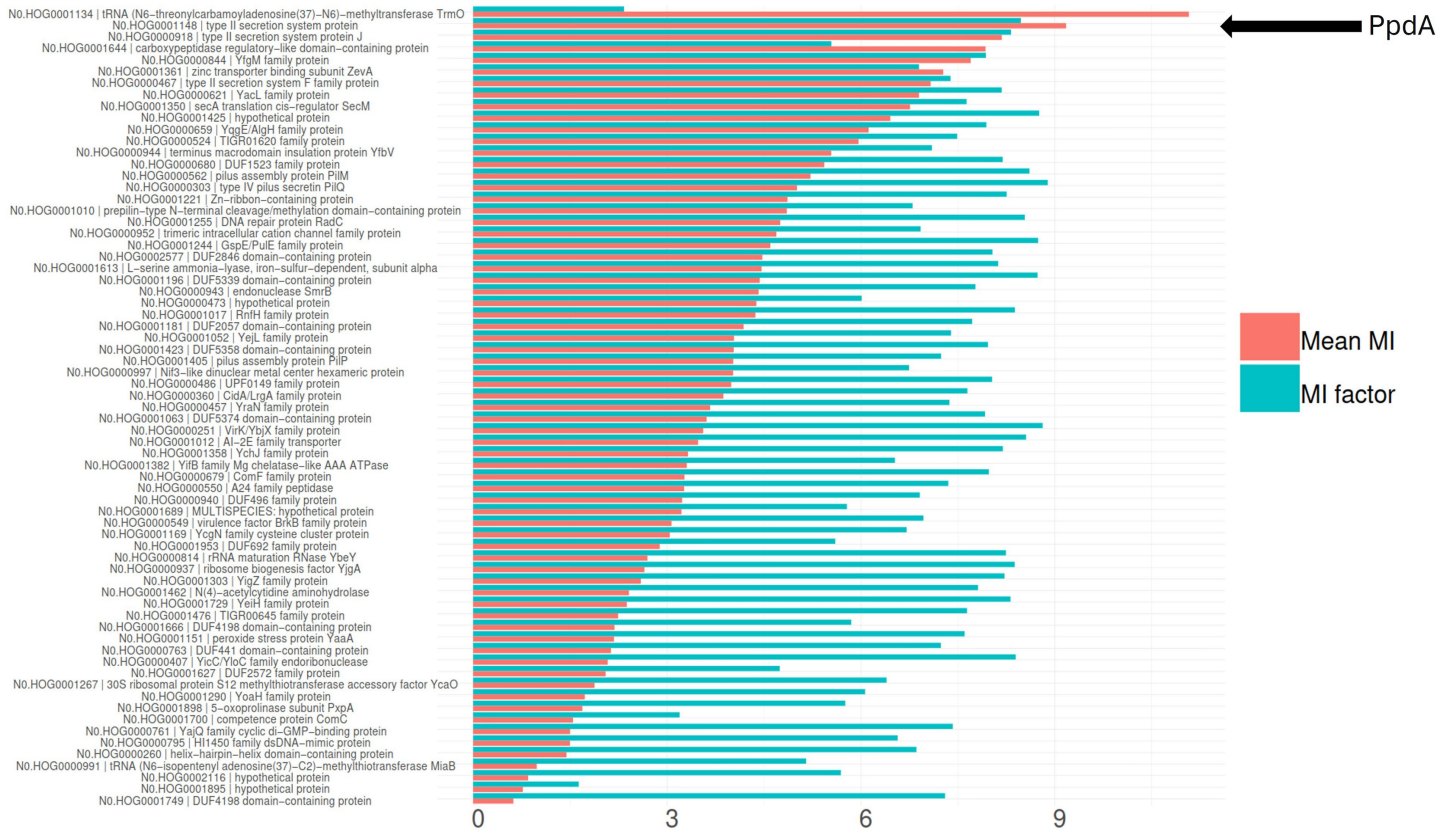
Coevolution of all modeled orthogroups and eUSS by miBIO. Combined bar plots showing mean MI and MI factor, sorted by mean MI. Mean MI was multiplied by 100 to better scale with the MI factor. OrthoFinder hierarchical orthogroup numbers and gene names are listed. The relative positioning of PpdA = N0.HOG0001148 type II secretion system protein is indicated by the black arrow.

To further explore the PpdA_Past_ candidacy as the USS-receptor, we looked for traces of reciprocal influence between orthogroups and eUSS in coevolution analyses. Φ*c* and MI were calculated for all pairwise orthogroup-eUSS positions. The 17-mer eUSS was used in the coevolution analysis to widen the evolutionary signal with variable DNA positions beyond the 9-mer core USS. Of all orthogroups tested, PpdA had the highest Φ*c* factor of 5.05 (Supplementary Figure S7; Supplementary Table S6), meaning the number of significantly correlated (*α* = 0.05) pairwise positions in the orthogroup and eUSS alignments (total number of significant Φ*c* values divided by the length of the sequences in the orthogroup). PpdA also ranked second in terms of total number of significant Φ*c* correlations (996), only exceeded by the DUF2057 domain-containing protein (1006) (Supplementary Figure S7; Supplementary Table S6). The YfgM family protein (YfgM) also had many significant pairwise Φ*c* correlations (961) with eUSS and a high (4.49) Φ*c* factor (Supplementary Figure S7; Supplementary Table S6). Other proteins with a combination of high correlation factor and number of significant correlations were SecA translation cis-regulator SecM (SecM) and terminus macrodomain insulation protein YfbV (Supplementary Figure S7 and Supplementary Table S6). Furthermore, PpdA was the second-ranking orthogroup in the miBio coevolution analysis regarding mean MI for positive MI values, surpassed only by tRNA N6-threonylcarbamoyl-adenosine(37)-N6-methyltransferase TrmO (Figure 4; Table 3), ranking ninth in terms of MI factor (number of positive MI values divided by length of the sequences in the orthogroup). We noted that the *trmO* gene (Hi_0510) has two intragenic *Hin-*USS in *H. influenzae* Rd., whereas *ppdA* (Hi_0938) has none (data not shown). Interestingly, PpdB, a protein predicted by STRING to be a functional partner of PpdA and also a minor pilin whose encoding gene is located adjacent to *ppdA/comN* in the *comNOPQ* operon (Supplementary Figure S6), ranked third in this analysis. YfgM family protein, which ranked second in the Φ*c* analysis, ranked fifth in the MI factor.

**Table 3:** The top 10 orthogroups in terms of mean MI in miBIO.

OrthoFinder HOG	OrthoFinder OG	Gene name	OrthoFinder coverage	Protein length	Mean non-negative MI
N0.HOG0001134	OG0000950	tRNA (N6-threonylcarbamoyladenosine(37)-N6)-methyltransferase TrmO	147	272	0.1108185209
N0.HOG0001148	OG0000964	type II secretion system protein	147	198	0.0918067493
N0.HOG0000918	OG0000734	type II secretion system protein J	148	283	0.0818427395
N0.HOG0000844	OG0000660	YfgM family protein	149	215	0.0770323591
N0.HOG0001361	OG0001177	zinc transporter binding subunit ZevA	137	270	0.0727758382
N0.HOG0000467	OG0000283	type II secretion system F family protein	149	434	0.0708391123
N0.HOG0000621	OG0000437	YacL family protein	150	147	0.0690436857
N0.HOG0001350	OG0001166	secA translation cis-regulator SecM	141	164	0.0676582310
N0.HOG0000659	OG0000475	YqgE/AlgH family protein	149	204	0.0612235445
N0.HOG0000524	OG0000340	TIGR01620 family protein	146	422	0.0596713961

Coverage refers to the number of OD species with orthologs in the orthogroup.

### PpdA-eUSS coevolution analysis

**Figure 5:**
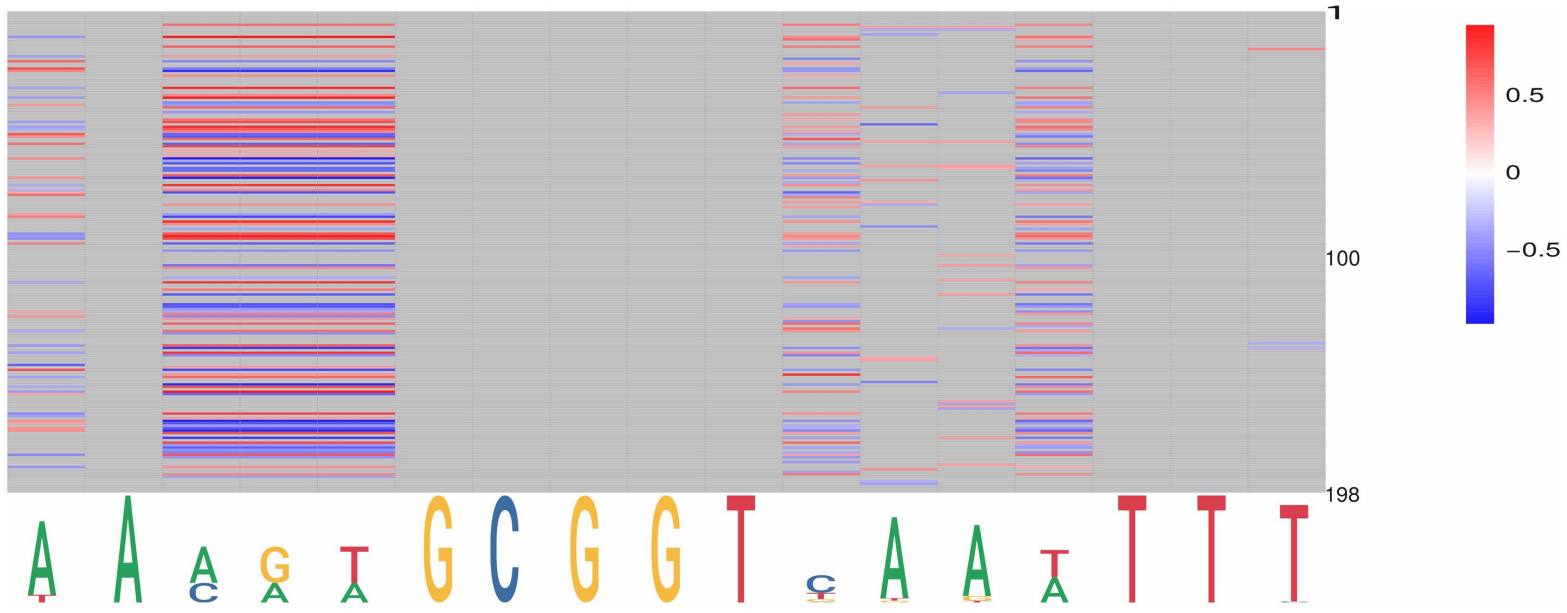
Graphical representation of the significant pairwise correlations for PpdA positions and eUSS by miBio. PpdA positions on the vertical axis (1–197) and eUSS positions (1–17) on the horizontal axis as a sequence logo with the 9-mer USS underlined. miBIO correlations according to the given scale (−0.5 to 0.37), with negative and non-correlating MI values in blue, neutral in white, and positive in red. Non-informative eUSS positions are gray.

**Figure 6:**
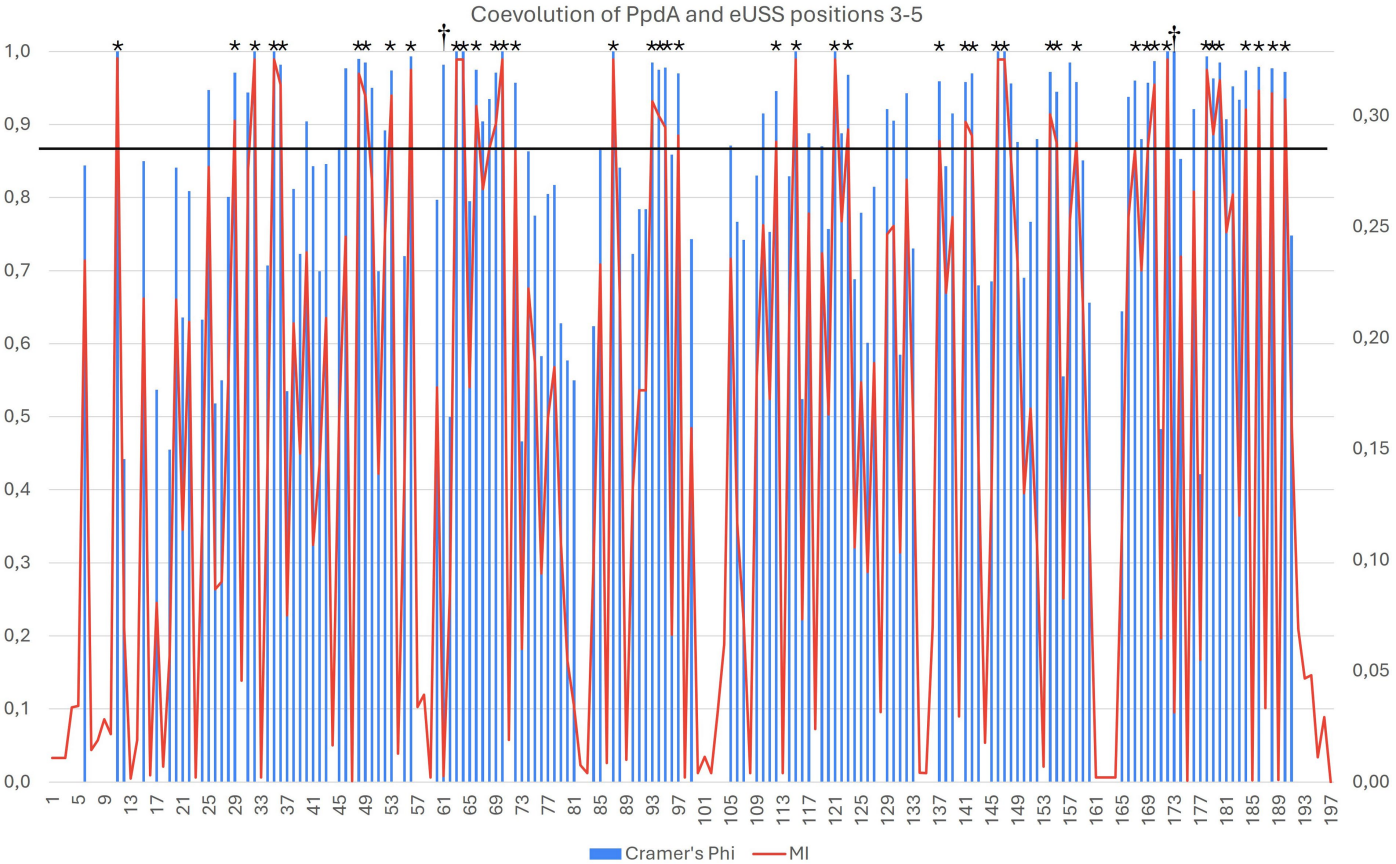
Overlap in the significant pairwise correlations for PpdA positions and USS dialect-specific eUSS positions 3–5 by Cramér’s Φ and miBio. Combined bar and line plot of Φ (blue bars) and MI (red line) values showing the variable degrees of coevolution across PpdA_Past_ to the three eUSS positions 3–5. The X-axis shows all the PpdA positions in the alignment and the Φ (left) and MI (right) values on the Y-axes. The horizontal line at Φ*_C_* > 0.9 and MI > 0.29 demarcates 42 particularly strong coevolved positions marked with *. Positions 94–96 and 122 represent indels distinguishing the PpdAs of *Apl*-USS and *Hin*-USS species, whereas the rest of the strongly coevolved positions represents informative amino acid variation. Positions 62 and 174 marked with † are positions where the Φ and MI have values at either end of their respective ranges.

By targeting PpdA in the coevolution analysis against other orthogroups, we further explored the coevolutionary signal in the Φ*_C_* and MI analyses of PpdA-eUSS (Supplementary Tables S7, S8). All PpdA numbering in these analyses refers to the numbering in the full-length PpdA_Past_ alignment in Supplementary data 1. This PpdA_Past_-alignment with per-position variable amino acids and indels paired with all eUSS positions in the 17-mer eUSS provided 996 unique pairs with variable coevolutionary signals. The pairwise sites that yielded the strongest positive MI values for PpdA-eUSS in miBIO showed considerable overlap with those in Φ*c*, and the overall distribution of positive MI values and significant Φ*c* were highly similar. As shown in Figure 5 and Supplementary Figure S8, particularly informative coevolved positions in the PpdA_Past_ alignment were found relative to eUSS positions 1, 3–5, 11, 13, 14, and 17 in both analyses. The inner GCGGT core in eUSS positions 6–10, which is well-conserved across the two USS dialects, showed, as expected, no significant coevolutionary signals alongside positions 2, 12, 15, and 16 outside of the 9-mer USSs. Since eUSS positions 3–5 distinguish the two USS dialects, we explored their coevolutionary signals in detail, observing a mix of conserved and coevolved positions across the whole PpdA. The two methods showed considerable overlap in their coevolutionary scores (Figure 6). 47 PpdA alignment positions with strong coevolutionary signals using both methods were identified. These positions included two indels and 43 amino acid variations, which could influence USS binding and USS dialect specificity. The informative positions were distributed across all domains of PpdA, making the identification of a singular domain candidate for the USS dialect differences a task for future pursuits. The results emphasize the phylogenetic distances between PpdAs of the *Apl*-USS and *Hin*-USS clades and a very limited extent of horizontal gene transfer of the *ppdA* gene between them.

### PpdA and PpdA_USS_ structural characteristics

**Figure 7:**
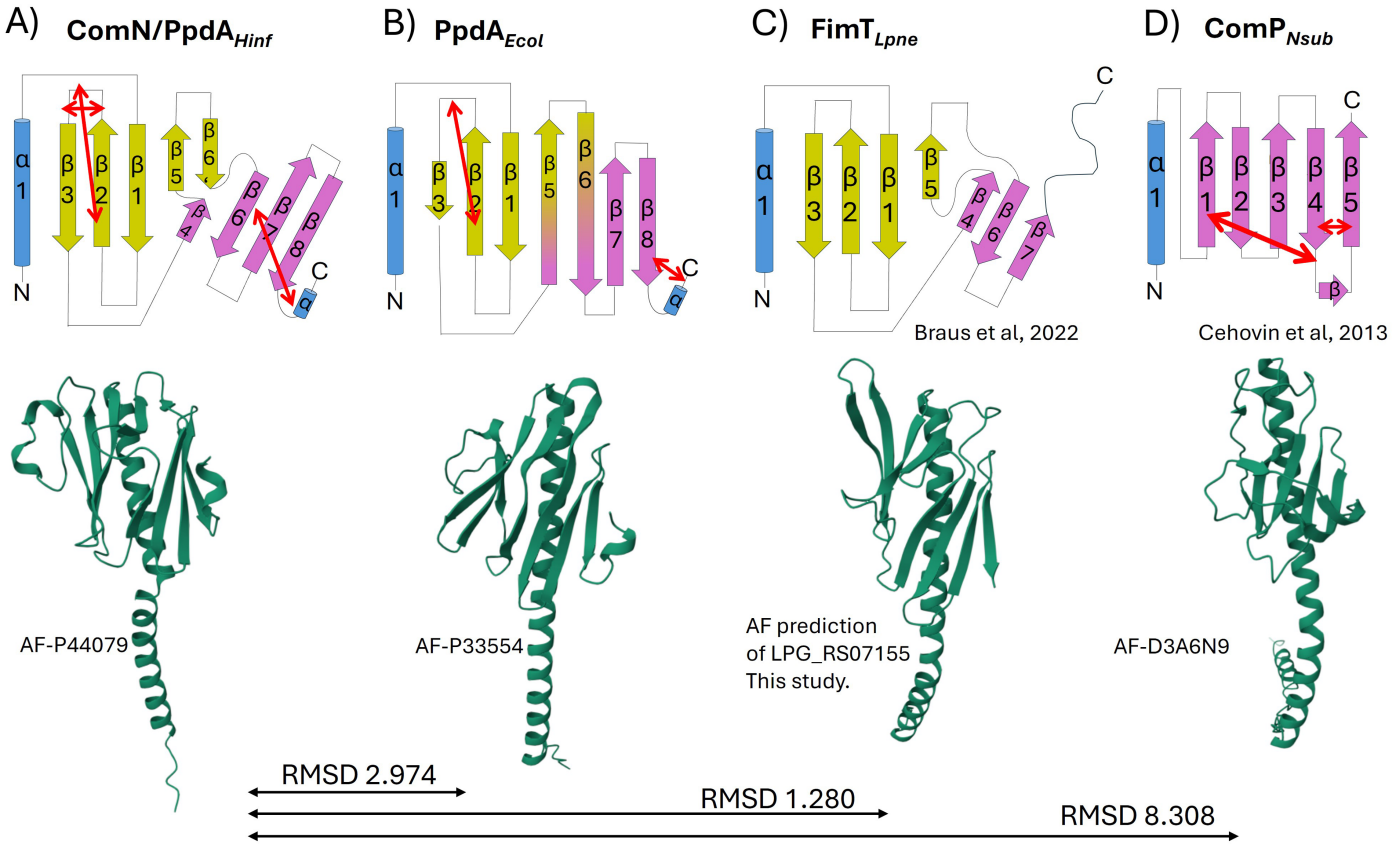
Topology diagrams and AlphaFold modeled structures of PpdA_Past_, PpdA*_Ecol_*, FimT*_Lpne,_* and ComP_Neis_. The common α-helix (truncated in the topology diagrams) at the N-terminal of the proteins is colored in blue, and β-sheets in yellow and purple. Short α-turns and β-strands are indicated. Disulfide bridges are shown with double red arrows. The topology diagrams and coloring are based on the FimT_Lpne_ described in Braus et al. (2022). The 3D ribbon diagrams are from AF with respective structure reference numbers. **(A)** PpdA_Past_ from *H. influenzae* with one N-terminal α-helix, eight β-strands, and three disulfide bridges. **(B)** PpdA of *E. coli* with one N-terminal α-helix, eight β-strands, and two disulfide bridges. **(C)** FimT of *L. pneumophila* with one N-terminal α-helix, seven β-strands, and no disulfide bridges. The ribbon diagram was obtained by folding FimT_Lpne_ in AF. **(D)** ComP of *N. subflava* with one N-terminal α-helix, five β-strands, and three disulfide bridges.

**Figure 8:**
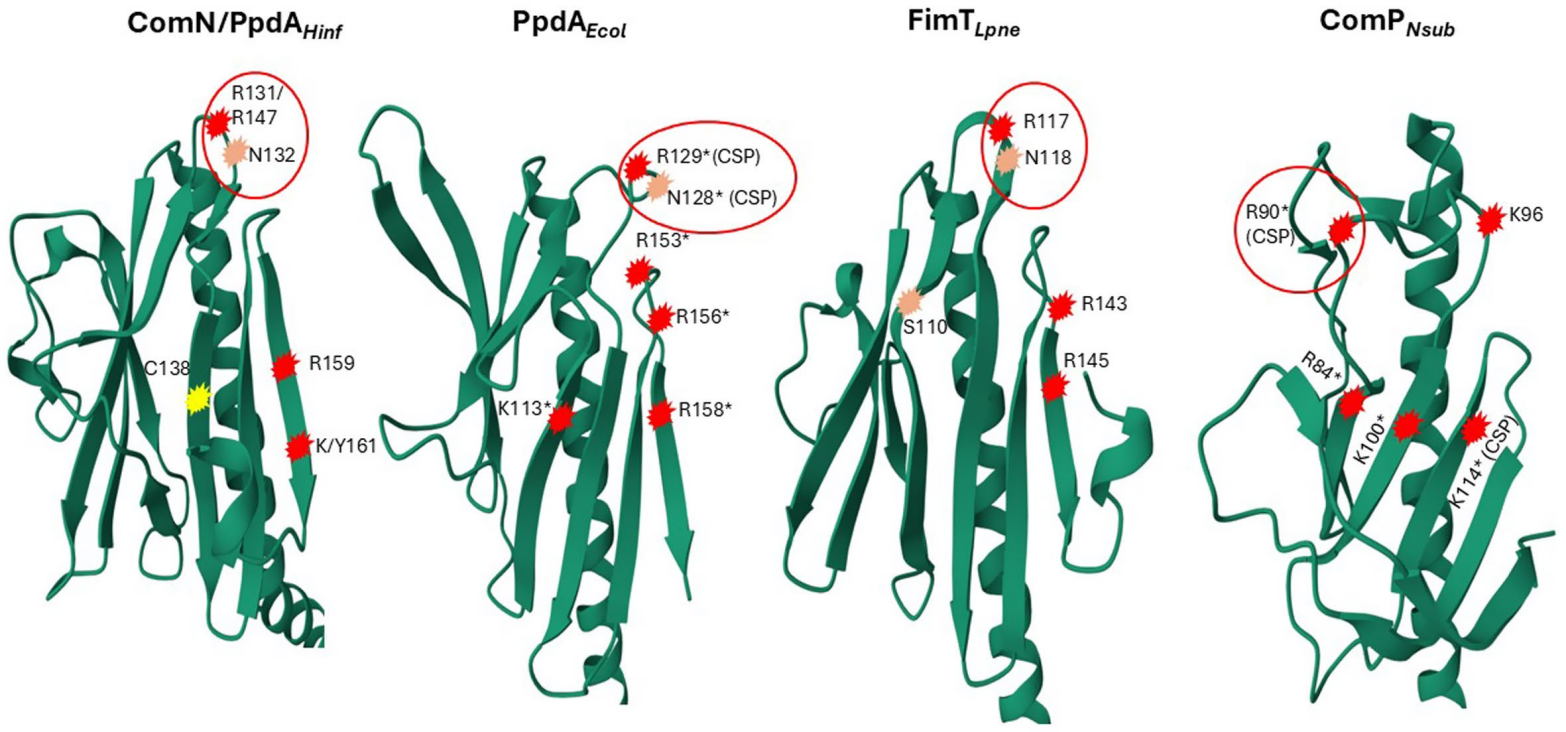
3D ribbon representations of PpdA*_Hinf_*, FimT*_Lpne_*, PpdA*_Ecol,_* and ComP_Neis_. Notably, positively charged and polar amino acids are encircled in red. R131/R147 in PpdA_Hinf_ refers to the *H. influenzae* Rd. full-length primary sequence and the PpdA_Past_ alignment position, respectively. Encircled R129 and N128 in FimT*_Lpne_* shown to give the strongest chemical shift perturbations (CSPs) upon DNA-binding in Braus et al. (2022) are marked with *(CSP) and single amino acid substitutions (R/K to Q) shown to affect negatively DNA-binding/transformation in an additive manner are marked *. Residues in ComP_Neis,_ which have been shown to produce significant CSPs upon DUS binding, and single amino acid substitutions (R/K to A), have been shown to affect DNA-binding/transformation negatively (Berry et al., 2016) are marked in the same way. Similarly located positively charged/polar amino acids in and above the C-terminal β-sheet are numbered and encircled in PpdA*_Hinf_*, and PpdA*_Ecol_.* C138 in PpdA*_Hinf_* connecting the C-terminal loop to β6 located in the same position as K113* in FimT*_Lpne_* is marked in yellow.

**Figure 9:**
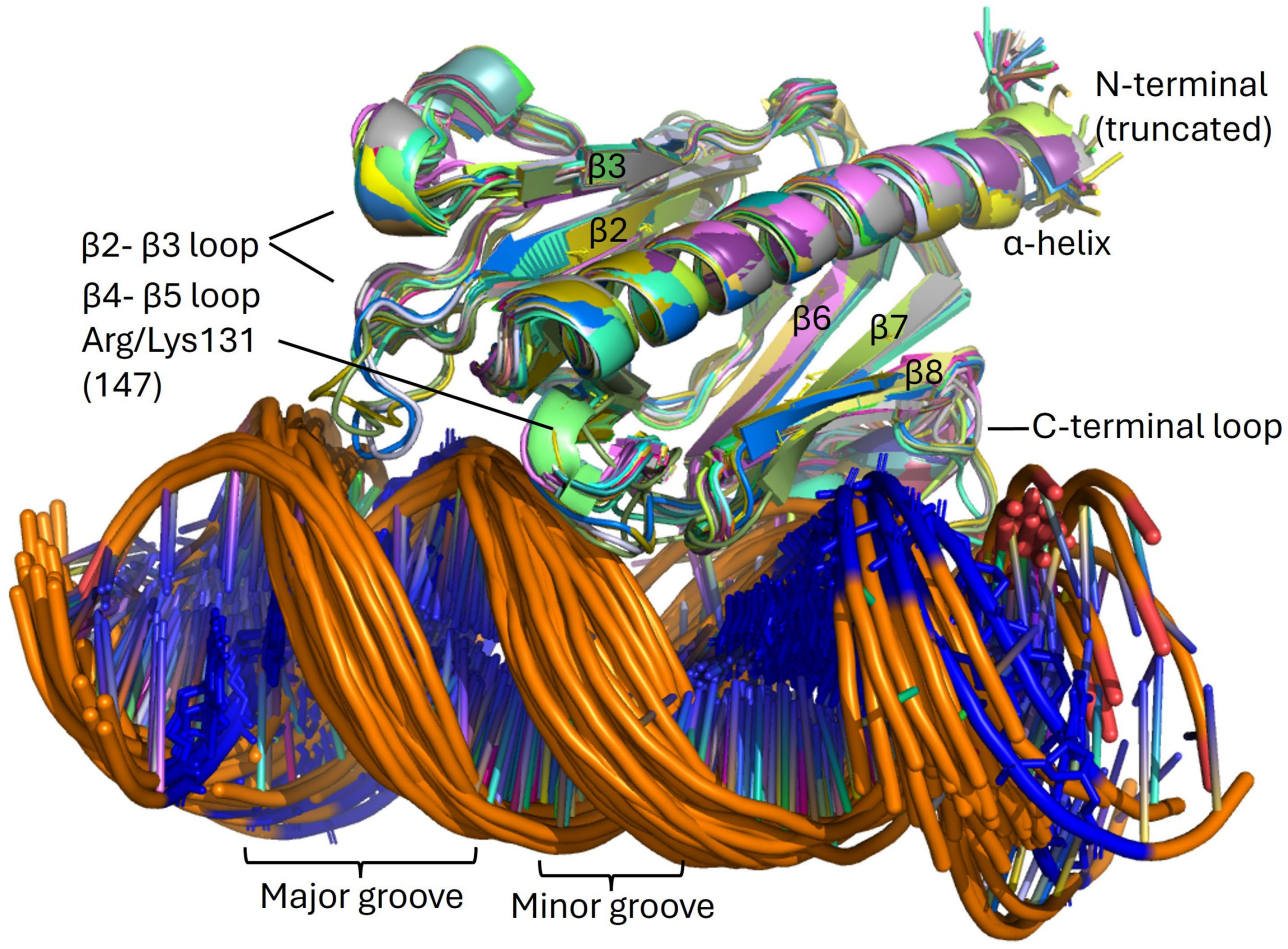
The PpdA_Past_-eUSS complex. Overlays of high confidence PpdA_Past_-eUSS complexes (*N* = 57) of the OD group as modeled by AF3. β-strands and β-β loops are assigned in addition to the grooves on DNA. USS-dialect-specific residues (AGT of *Hin-*USS and CAA of *Apl-*USS) are shown in blue coloring in the DNA, showing DNA-binding in two opposite orientations.

**Figure 10:**
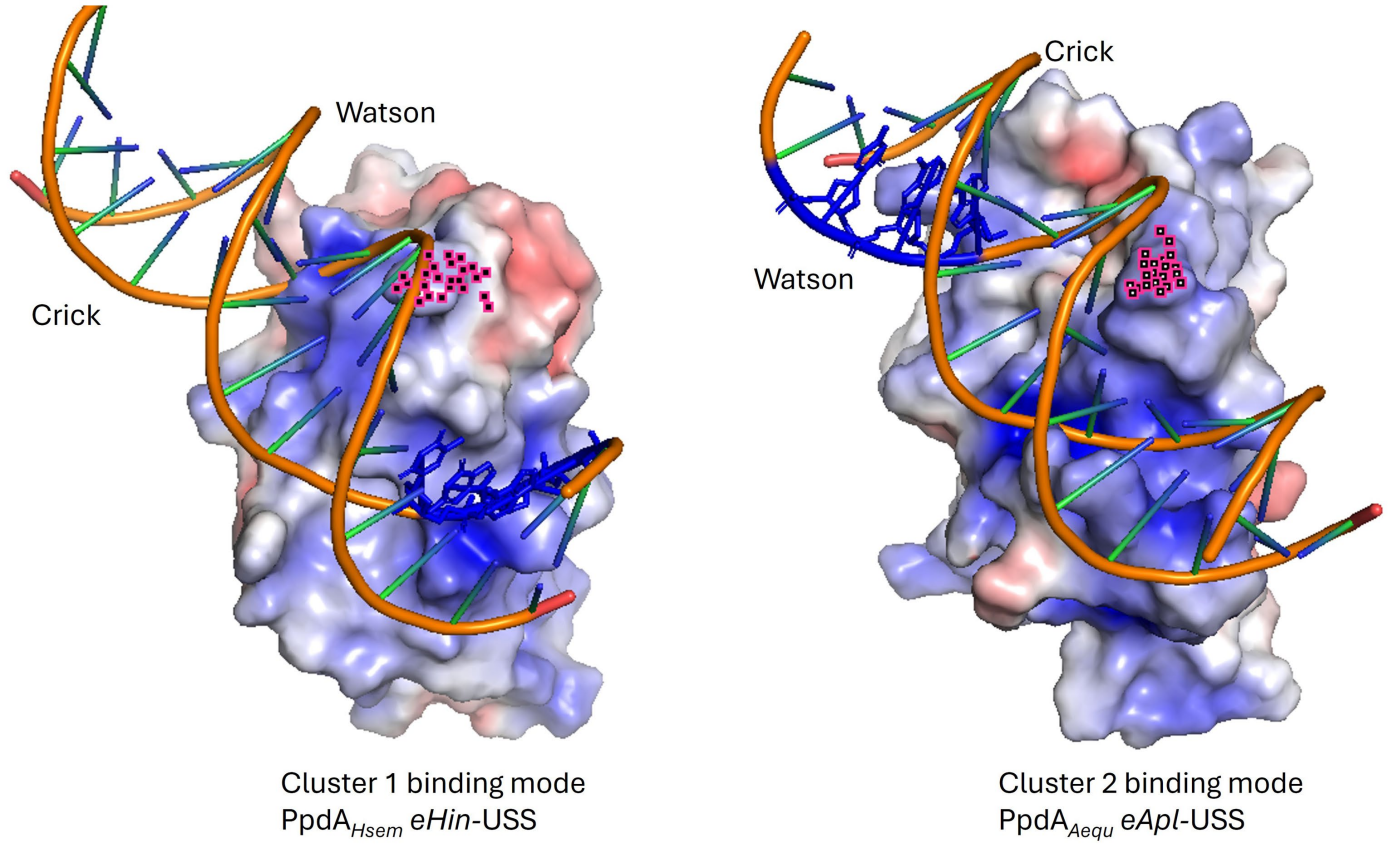
Electrostatic maps of representative PpdA_Past_-eUSS binding modes. Illustrative examples of the two main PpdA-eUSS binding modes displaying their opposite USS orientation. In cluster 1 (ex. *H. seminalis* SZY H68) the Watson strand enters C-terminal β-sheet surface delimited by the C-terminal loop and a special Arg whose atoms are colored in pink, interacts with the Crick strand in a manner opposite to cluster 2 (ex. *Actinobacillus equuli* subsp. haemolyticus strain 3524). USS-dialect-specific residues (AGT of *Hin-*USS and CAA of *Apl-*USS) are shown in blue coloring in the DNA. *Hin*-USS-dialect PpdAs were modeled in both clusters 1 and 2 and *Apl*-USS PpdAs exclusively in the cluster 2 USS orientation.

**Figure 11:**
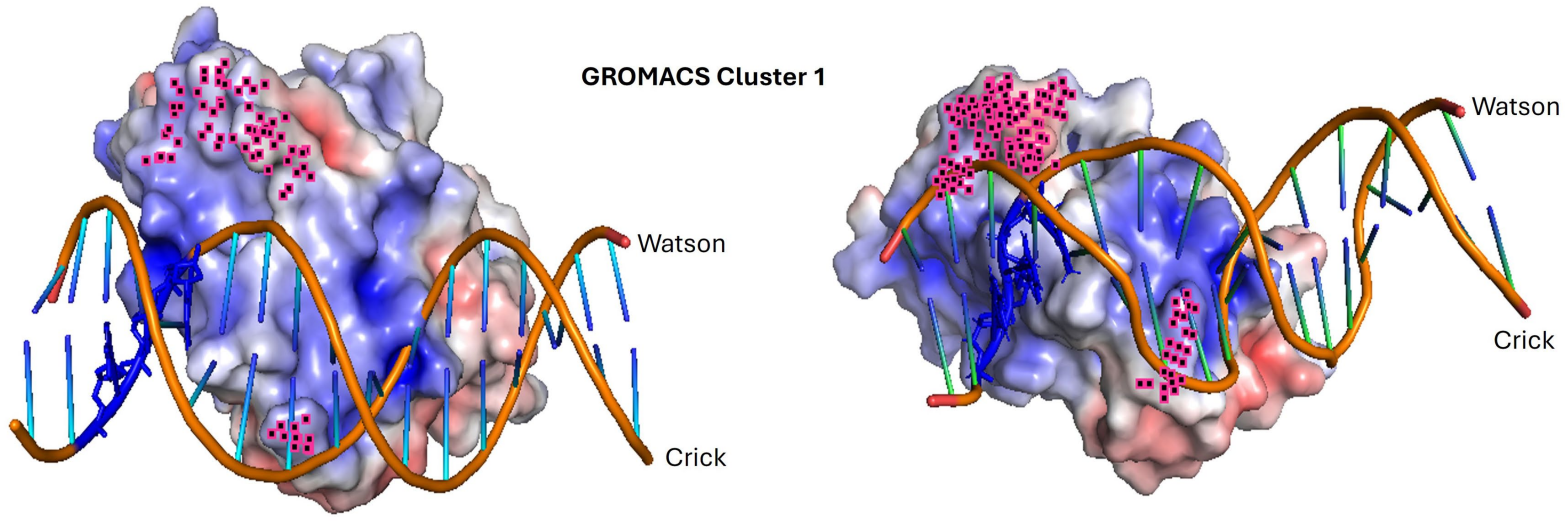
Molecular dynamics simulation of the highest confidence representative in cluster 1. The dynamic interaction between PpdA*_Hsem_* (*Haemophilus seminalis* SZY H68) and 17-mer *Hin*-eUSS. The input structure is shown to the left, and the resulting structure to the right. The penultimate 9 C-terminal amino acids and R131 are highlighted in pink. *Hin*-USS-specific nucleotides are shown in blue. Both protein and DNA shapes have changed during the molecular simulation and seem to have brought the USS-dialect-specific nucleotides more tightly into the electropositive pocket over the C-terminal β-sheet. The less conserved AT-rich region beyond the 9-mer core USS is the DNA region, which is the least integrated part of the structure.

Having found robust modeling support for *Pasteurellaceae* PpdA as the prime USS-receptor candidate above, we explored the amino acid sequence alignment of PpdA orthologs from Pasturellaceae highly enriched with *Apl-*USS (*Frederiksenia*, *Mannheimia*, and *Actinobacillus*) and *Hin-*USS (*Haemophilus* and *Aggregatibacter*) (Supplementary Figure S9), their common topology (Figure 7A), 3D structure, and AF3 modeled DNA-binding mode (Figures 8–11). All PpdA_Past_ were found to contain six conserved Cys residues (Cys76, Cys87, Cys89, Cys95, Cys138, and Cys169 in PpdA*_Hinf_* full-length numbering) (Supplementary Figure S9). PpdA clustered in distinct groups adhering to taxonomic genera divisions and notably also to USS-dialect (*Apl-* and *Hin-*) specificity. The PpdAs of the *Apl-*USS group were locally found to be 3–5 amino acids longer than the two *Hin-*USS groups, with mostly polar residues in the long β2-β3 loop (Supplementary Figure S9). The *Haemophilus* group contained a unique Lys111 in the β3-β4 loop. The secondary and tertiary structure of PpdA*_Hinf_* in Figure 7A shows how the protein adopts a classic minor pilin structure consisting of an N-terminal α-helix and a large globular domain consisting of two distinct β-sheets, one N-terminal and one C-terminal. The β1-strand is located across the center of the globular domain of the N-terminal β-sheet, and the β2-β3 loop forms a relatively large and complex domain. The β4 and β5 strands are short and unidirectional, completing the two β-sheets with the also unidirectional β6’ and β6 strands. The six Cys residues form three disulfide bridges, which fold the β2-β3 loop on itself and onto β2 and the C-terminal loop onto β6, notably transversing β8 and β7.

As an objective measure of structural similarity between different PpdA_Past_, the root mean square deviation of atomic positions (RMSD) was found to be <1.079 across PpdAs from phylogenetically distant species with different USS-dialects (*Hin*-USS reference *H. influenzae* Rd. PpdA vs. respective PpdAs in *Apl*-USS species *Aggregatibacter* sp. oral taxon 513, *F. canicola* HPA21, and *Mannheimia bovis 39324S-11*) and <0.440 between *H. influenzae* Rd. and *Actinobacillus equuli* subsp. haemolyticus strain 3,524, with the same *Hin*-USS specificity. The overall structure of PpdA_Past_ resembles that of *E. coli* PpdA (PpdA*_Ecol_*) (RMSD <3 Å) and *L. pneumophila* FimT (FimT*_Lpne_*) (RMSD <1.3 Å) and shares features with Neisseriaceae ComP (ComP_Neis_) (RMSD 8.308) (Figures 7A–D, 8; Supplementary Figure S10).

Like PpdA_Past_, PpdA*_Ecol_* adopts a classic minor pilin structure consisting of the N-terminal α-helix and the large globular domain consisting of two distinct β-sheets. The amino acid coverage and sequence identity of PpdA*_Hinf_* and PpdA*_Ecol_* were 88 and 18.3%, respectively, with an RMSD of 2.974 Å, showing that they are closely homologous proteins (Reva et al., 1998; Supplementary Figure S15). Notable conserved residues were Cys79, Cys89, and Cys172 (PpdA*_Hinf_* full-length numbering), a Leu-aromatic-Leu-Arg motif in the α-helix, Asn60 at the C-terminal end of the α-helix, a Trp-Cys-Leu motif in β2, a Gly-branched hydrophobic-Arg-Asn-Thr motif on the β5-β6 loop, and conserved Arg162 in β8. Prolines on both sides of β3 and β4 also align, of which there are two in the β3-β4 loop. The β1 is also located across the center of the globular domain, and the β2-β3 loop similarly forms a relatively large complex domain. PpdA*_Ecol_* was modeled to share with PpdA*_Hinf_* the disulfide bridge connecting the β2-β3 loop to β2, whereas the C-terminal part was bridged adjacently to β8 in PpdA*_Ecol_* and not in a β-sheet transversing manner to β6 as in PpdA*_Hinf_*. The β4, β5, β6’, and β6 of PpdA*_Hinf_* were modeled as long continuous and twisted β-strands in PpdA*_Ecol_* to dually complete the two β-sheets, similarly to how the short discontinuous β-strands of PpdA*_Hinf_* were modeled.

As previously shown by Braus et al. (2022) and the modeled structure of locus tag LPG_RS07155 by AlphaFold, FimT*_Lpne_* also adopts a classic minor pilin structure consisting of the N-terminal α-helix and the PpdA-similar large globular domain consisting of two distinct β-sheets. The amino acid coverage and sequence identity of PpdA*_Hinf_* and FimT*_Lpne_* were 73.5 and 15.5%, respectively (data not shown), and an RMSD of 1.280 Å. Like PpdA*_Hinf_* and PpdA*_Ecol_*, the β1 strand is also located across the center of the FimT*_Lpne_* globular domain, demarcating the two β-sheets. The unstructured and long C-terminal domain is folded by AF3 into the β8 strand, unlike the previously published topology map and NMR structure (Braus et al., 2022). The β2-β3 loop is comparatively small and does not fold over β2 as in PpdA*_Hinf_* and PpdA*_Ecol_*. FimT*_Lpne_* has no Cys residues and hence no disulfide bridges. The third last C-terminal amino acid of FimT*_Lpne_*, a Gly, was modeled by AF to electrostatically connect β8 to β7. The combined loss of two (Arg146/Arg148) or three (Arg143/Arg146/Arg148) positively charged amino acids in the β7-β8 loop and in the β8 strand has been shown to reduce the DNA-binding affinity of FimT*_Lpne_* 10-fold or more (Braus et al., 2022). Two Arg residues (Arg143 and Arg145) are conserved in similar positions in PpdA*_Ecol_*, one in the β7-β8 loop and one in the β8 strand (Figure 8; Supplementary Figure S10), suggesting that they could be involved in non-specific DNA-binding. PpdA*_Hinf_* has one Arg and one Lys in β8 and could therefore also potentially function in DNA-binding. All USS-rich Pasturellaceae PpdAs explored in depth here (*Haemophilus, Fredriksenia, Actinobacillus, Mannheimia*, and *Aggregatibacter*) have several positively charged residues in or in conjunction with β8 on the C-terminal side of the last Cys in the C-terminal loop. The exact positioning of these positively charged residues follows the USS-dialect division of the *Pasturellaceae* PpdAs (Supplementary Figure S9), and their potential role in DNA-binding and DNA-binding specificity should be further explored experimentally using previously established *in vivo* and *in vitro* approaches (Cehovin et al., 2013; Berry et al., 2016; Berry et al., 2019). Furthermore, Braus and colleagues (Braus et al., 2022) showed that the highest chemical shift perturbations (CPSs) upon 12-mer dsDNA-binding were found for residues Asn118 and Arg119 in the β5-β6 loop of FimT*_Lpne_*. These two amino acids are conserved as Arg-Asn in PpdA*_Ecol_*, PpdA*_Hinf_* and in genus *Aggregatibacter* PpdAs, as Gly-Arg-Leu in genera *Frederiksenia* and *Actinobacillus* PpdAs and as Ser-Gly-Gln in genus *Mannheimia* PpdAs (Supplementary Figure S9). The combined polar Ser and Gln in the latter lacks the long-range electrostatic pull of Arg/Lys, but may also potentially bind to DNA through H-bonds (Luscombe, 2001).

The only minor pilin with experimentally established DNA-binding specificity is ComP in the *Neisseria* (Cehovin et al., 2013; Berry et al., 2016; Berry et al., 2019). ComP differs from PpdA*_Hinf_*, PpdA*_Ecol,_* and FimT*_Lpne_* by having a single β-sheet of five strands interconnected with two disulfide bridges. The two disulfide bridges in ComP are essential for DUS-specific binding (Cehovin et al., 2013). One of these connects a long C-terminal loop (DD-loop) in a transversing manner across the β-sheet onto the β1 strand (Figure 7D). In the DUS-specific ComP*_Nsub_*, the highest CPSs upon DUS binding were found to be in the β1-β2 loop containing its central Lys/Arg90 (Berry et al., 2016). Like Arg119 of FimT*_Lpne_* is positioned above the N-terminal part of the C-terminal β-sheet, Arg90 of the *Neisseria* ComP is also positioned above the N-terminal part of its single β-sheet and could indicate similar and possibly initial roles in DNA-binding. We have recently described two differently modeled DUS binding modes of Neisseriaceae ComPs, and in both binding modes are the Lys/Arg90 and the DD-loop modeled to interact with grooves of the DNA (Helsem et al., 2025). PpdA*_Hinf_* similarly connects its C-terminal α-loop across the β-sheet to the β6 strand. In the AF3 top-scoring PpdA_*Hin*-USS_ models and other PpdA_USS_ and PpdA_eUSS_ models, this C-terminal loop and the Arg-Asn motif are modeled to interact with DNA (Figure 9; Supplementary Figures S10, S11).

AF3 modeled with the highest confidence PpdA_eUSS_ complexes showing the first part of the β2-β3 loop and the C-terminal loop to interact with the minor groove one helical turn apart and the short β4 and β5 strands to interact with the major groove (Figures 9, 10). In exploring the PpdA DNA-binding mode we observed the majority of all modeled complexes adopted the same DNA groove interactions in both USS and eUSS models. However, the 17-mer eUSS models were split into two clusters numbered 1 and 2, characterized by either of two 180° opposite USS orientations (Figure 10). Mapping coevolved amino acids onto these different PpdA-eUSS clusters was explored to shed some light on which may be best supported (Supplementary Figure S11). Coevolved residues modeled in proximity to eUSS in cluster 1 showed how the USS-dialect differing positions 3–5 were positioned above the C-terminal β-sheet and the strongly coevolved C-terminal loop. The 9-mer USS models were all as cluster 1 of the eUSS models showing USS-dialect-specific nucleotides buried into an electropositive patch in PpdA in the C-terminal β-sheet region.

**Figure 12:**
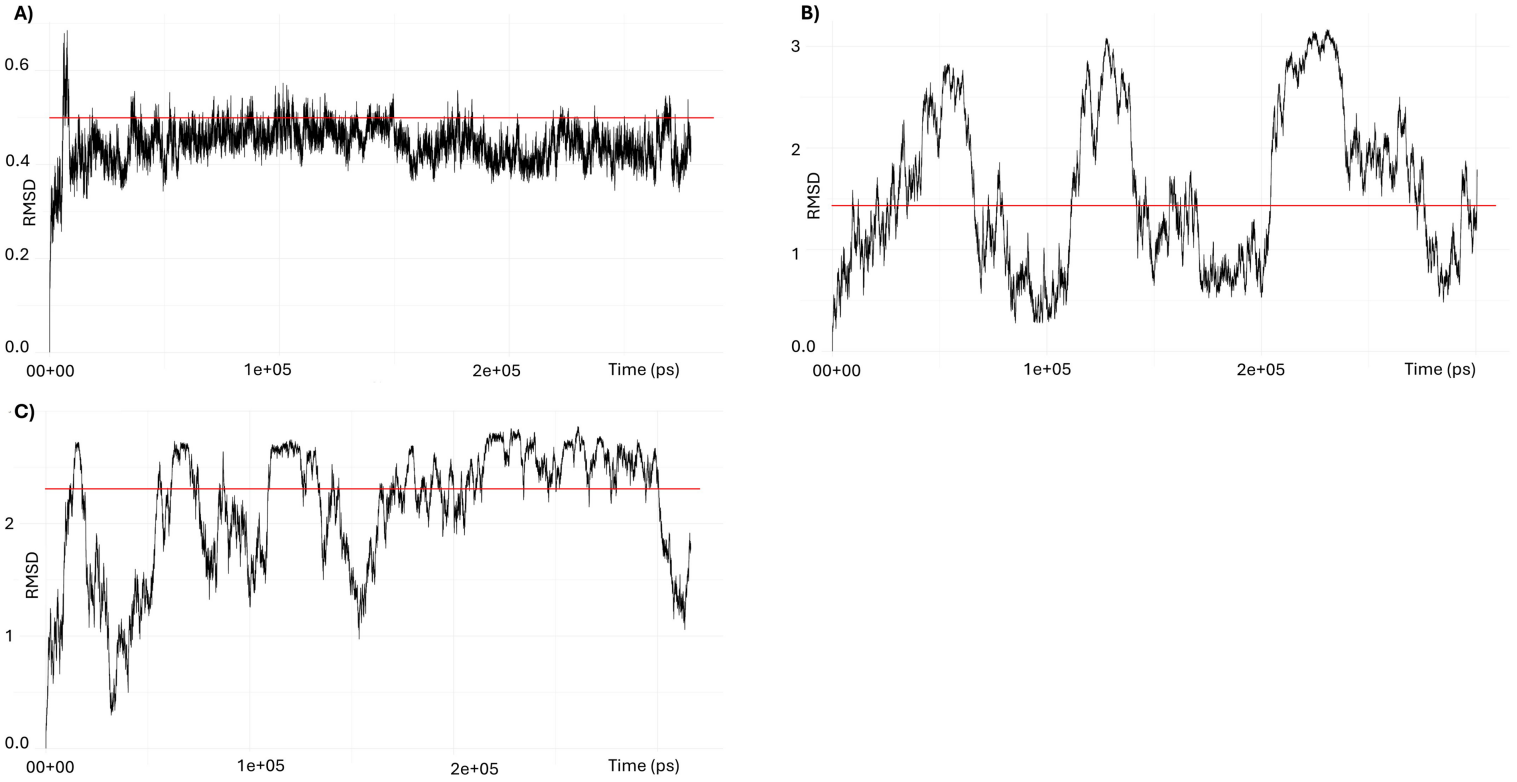
RMSD trajectories in GROMACS simulations of PpdA-USS complexes. **(A)** RMSD of the 9-mer USS-PpdA complex over a 300-ns molecular dynamics simulation. After an initial equilibration phase, the complex reached a stable plateau oscillating around a median of 0.44 Å, indicating a highly stable configuration throughout the trajectory. **(B)** RMSD of the 17-mer eUSS-PpdA complex of cluster 1 over a 300 ns molecular dynamics simulation. The RMSD fluctuates throughout the trajectory with amplitudes up to 2.5 Å, but remains consistently below 3 Å. The median RMSD of 1.46 Å suggests that the complex is stable overall, despite moderate dynamic conformational flexibility in the bound state. **(C)** RMSD of the 17-mer eUSS-PpdA complex of cluster 2 over a 300 ns molecular dynamics simulation. After initial equilibration with >2.5 Å amplitudes the trajectory shows a converging trend toward a plateau around 2.5 Å during the second half of the run, yet with downward fluctuations to 1 Å, indicating a stable bound configuration with moderate conformational flexibility.

Molecular dynamics simulations of PpdA_USS_ and PpdA_eUSS_ complexes using GROMACS (Figures 11, 12; Supplementary Figures S12, 13) showed that only models with the core 9-mer USS reached a stable state, where RMSD oscillated around the median 0.45 Å, indicating that the DNA-binding interaction was confidently preserved (Figure 12A). The eUSS PpdA simulations of cluster 1 (Figures 11, 12B) and cluster 2 (Figures 12C and Supplementary Figure S13) showed moderately stable configurations oscillating RMSDs within 1 and 3 Å. The median RMSD of Cluster 1 was 1.46 Å and 2.29 Å for Cluster 2. Together, the simulations showed that cluster 1 and the 9-mer USS models were of lower median RMSD than cluster 2 PpdA_eUSS_ models. Comparing the RMSD plots of clusters 1 and 2 indicated that cluster 1 was better at finding stable structures closer to the input structure below 2 Å for prolonged periods of time and that cluster 2 showed some plateauing behavior above 2 Å.

## Discussion

### Protein-DNA modeling support of PpdA-USS

In searching for the USS-receptor, 293 Pasteurellaceae orthogroups were identified (eggNOG) using the *H. influenzae* Rd. genome, as reference. *H. influenzae* Rd. was the first bacterial genome to be fully sequenced (Fleischmann et al., 1995). It was considered the most fitting reference since almost all experimental uptake and transformation data in Pasteurellaceae are from this species and strain. The wild-type phenotype of *H. influenzae* Rd. displays very high USS-specific transformation frequencies upon cAMP competence induction (Sinha et al., 2020), documenting the presence of both an expressed and highly specific USS-binding protein. The 293 orthogroups were filtered based on functional annotations and orthogroup coverage in the Pasteurellaceae and reduced to 73 orthogroups in the 73O dataset which were considered exhaustive in being based on rich GO annotations and functional terms known to be relevant for DNA uptake, transformation and competence. We expected that Pasteurellaceae family members, at least those with genomes highly enriched with USS, would have USS-specificity encoded by a surface exposed protein constituting a part of current transformation models (Dubnau and Blokesch, 2019) including that of *L. pneumophila* (Braus et al., 2022) which is also a γ-proteobacterium. It has also been firmly established in DNA-binding and uptake studies that USS-binding takes place on the extracellular side of the outer membrane (Mora et al., 2020), warranting the focus on proteins and protein domains facing this environment. PpdA was the only orthogroup that consistently yielded AF3 models of protein-DNA complexes of high quality (high ipTM and pLDDT; low PAE and CPPM). Furthermore, PpdA was the only protein that yielded high-quality models in all ten modeled Pasteurellaceae species. Moreover, this orthogroup was the only one for which orthogroup_USS_ models had significantly higher ipTM and pLDDT and significantly lower CPPM than orthogroup_scr_ models. Since both PpdA_USS_ and PpdA_scr_ complexes were of low PAE and non-significantly different, it suggests that one non-sequence specific DNA-binding mode(s) can be repeatedly and robustly modeled on the PpdA structure by AF3. The lower CPPM of PpdA_USS_ than PpdA_scr_ complexes shows that AF3 is better at modeling complexes with minimum PAE across all nucleotide-amino acid residue pairs between DNA and protein chains when USS is used. Together we find that the AF3 results alone convincingly target PpdA as a DNA-binding protein and importantly the USS receptor in Pasteurellaceae. The only previous study to search for the USS-receptor (Molnar, 2004) characterized six other genes/proteins as plausible USS-specific candidates in addition to ComN, which are included in our 73O dataset [HI0436 (*comD*); HI0438 (*comE*); HI0299 (*pilA*), HI0939 (*comO*), HI0940 (hypothetical), HI0941 (hypothetical)], yet none of our AF3 models of these were found to be within ipTM_r_. Overall, the scarcity of high-quality AF3 models for non-PpdA proteins, in contrast to the robust support for high-quality models for PpdA, supports PpdA as the USS receptor.

### Predicted DNA-binding sequence specificity support for PpdA

The results from AF3 and DeepPBS were in agreement in that AF3 consistently modeled PpdA_USS_ with high confidence, while DeepPBS predicted specifically the two *Hin*- and *Apl*-USS dialects as binding motifs. It is important to note that potential artifacts in the AF3 results are likely to have been echoed downstream by DeepPBS, as both are neural networks and have similarities in architecture (personal communication with DeepPBS developers). Yet, the convolutions in DeepPBS are applied to separate DNA (Sym-helix) and protein entities, which potentially make the DeepPBS results more independent from the robust AF3 models used. Although some overlap in models and predictions cannot fully be excluded, we consider these convergent results to be strong lines of support for PpdA being the USS receptor. The DeepPBS results find additional support in the comparison of the DeepPBS predictions and the genome-wide *Hin-*USS conservation, as shown in the sequence logos for each species in Supplementary Figures S4, S5. We found informative cases where the weakest DeepPBS predicted USS positions were also the least conserved in each PpdA’s respective genome. One example is the uniquely underrepresented USS position 9(T) in *H. influenzae* Rd. predictions that matches the uniquely less conserved 9(T) in the *H. influenzae* sequence conservation logo. The other four species’ PpdAs had considerably stronger 9(T) predictions than *H. influenzae* Rd., and this was also reflected in their USS conservation. Other examples are described in Supporting Information. In the *Apl-*USS group, however, no apparent matches between USS conservation and predictions were observed. We speculate that the reason for not being able to identify similar correlations in the *Hin-*USS group is that the *Apl-*USS is considerably less conserved overall, with weaker conserved USS positions 1(A) and 2(C), and that the magnitude of their genomic enrichment is significantly lower (ca. 200–500 *Apl*-USS/Mb) than in the *Hin-*USS group (500–800 *Hin-*USS/Mb) (Supplementary Table S2). Also, specific uptake of *Apl-*USS in *Actinobacillus pleuropneumoniae* (an *Apl-*USS species) has previously been shown to increase only 17-fold relative to scrambled USS which is in stark contrast to the >1,500 fold increase in *Hin-*USS specific uptake in *H. influenzae* (Redfield et al., 2006). These observations could suggest that USS specificity is considerably less pronounced in the *Apl-*USS group of species than in the *Hin-*USS group and explain our difficulties with identifying matches between the PpdA_*Apl-*USS_ DeepPBS predictions and *Apl-*USS conservation. Further laboratory experiments on *Apl-*USS specificity are needed to explore *Apl-*USS specificity in more detail and in a wider group of Pasteurellaceae bacteria where *Apl-*USS is overrepresented.

### Coevolutionary support of PpdA

Having found strong support for PpdA as the USS receptor from modeling and sequence specificity predictions, it did not seem coincidental that PpdA also had the highest correlation factor in the Cramér’s Φ coevolution analyses of proteins and eUSS. The coevolutionary signal was higher by a Φ*c* correlation factor of 0.56 compared to the YfgM family protein (SecYEG translocon-subunit), while PpdA came second to a methyltransferase in the miBIO analysis. These results show that PpdA, if not itself responsible for shaping USS-specificity and genomic USS enrichment, at least has coevolved alongside USS-specificity in deep time and importantly through the divergence of USS specificity into distinct dialects. A caveat with the coevolutionary analyses is that coevolutionary signals may be caused by genetic drift aligning with the phylogenetic relationships in the input sequences. While the shuffling method in miBIO corrects for much of the phylogenetic drift, it remains a challenge to assess impact on coevolution particularly since the two USS dialects adhere to phylogeny. It is also possible that other proteins than the USS receptor also involved in the uptake process are imbued with coevolutionary relationships with (e)USS. For example, several of the type IV pilus machinery components (PilM, PilP, PilQ, ComF, and other minor pilins than PpdA) among the coevolved orthogroups show these relationships, yet with weaker coevolutionary signals than PpdA. Furthermore, due to the dependency between the USS-receptor and genomic enrichment of USS, several proteins would be in co-evolutionary relationships with the USS-receptor and hence also indirectly with (e)USS. The SecA translation cis-regulator SecM and YfgM (SecYEG translocon subunit), with high correlation values in our analyses, are such examples in that all pilins involved in USS-uptake specifically and non-specifically are dependent on Sec machinery for translocation (Kaushik et al., 2022). SecM has been shown to upregulate the functionality of SecA (Nakatogawa et al., 2004), to which *ppdA* and other pilins have adapted their prepilin signal peptide. Both the Φ*c* and miBIO results show co-evolution between eUSS and several of the other type IV pilus proteins, which are necessary for USS-mediated transformation. Since PpdA has the highest coevolutionary scores of these Type IV pilus machinery components, it supports its USS-receptor candidacy relative to the other type IV pilus proteins generally and to pilins specifically. An additional caveat is that regions of the genome with high eUSS content may disproportionately contribute to transformation, potentially generating spurious coevolutionary signals due to physical linkage between the eUSS and neighboring coding sequences or CDS rich in eUSS. The top-scoring miBIO TrmO may be an example of this co-evolutionary process since the *trmO* CDS is enriched with two eUSSs in contrast to none in *ppdA* genes themselves. These signals may reflect transformation-driven linkage disequilibrium rather than true evolutionary coupling between residues or sites. In these cases, the partitioning of *Hin-*USS and *Apl-*USS will thus closely follow the phylogeny and cause an inflation of the coevolutionary signal between these proteins and USS. Further studies of functional genomic USS enrichment could shed further light on this mode of evolution (manuscript in prep.). All these problems could result in falsely high positive Φ*c* and MI values in the coevolutionary analyses. However, we acknowledge that all proteins tested are faced with these challenges and thus the coevolution results are still helpful in pinpointing likely candidate USS receptor proteins. Perhaps even more so, the coevolution results tell us which proteins are likely *not* the USS receptor, as a high coevolutionary signal would be expected for the true USS receptor having shaped USS-rich Pasteurellaceae genomes in deep time.

### Gene regulatory and previously established phenotypic support of PpdA

The PpdA*_Hinf_* (HI0938) is identical to the previously assigned ComN protein, whose encoding gene constitutes part of the competence regulon in *H. influenzae*, designated as *comN* (Sinha et al., 2020; Molnar, 2004). This finding connected the modeled PpdA_Past_ orthogroup directly to competence for transformation to further strengthen its USS-receptor candidacy. The first *comN* knock out was made in *H. influenzae* (Molnar, 2004) and the loss of competence in this knock out strain was documented. However, this study could not exclude that the competence loss in Δ*comN* was due to a polar effect on the downstream genes in the same operon (*comNOPQ*). These limitations of the first Δ*comN* strain were later amended by the same group using in-frame deletions of all Sxy-regulated genes in the competence regulon, including *comN*, and confirmed the loss of DNA uptake and transformation in the single *comN* mutant (Sinha et al., 2020). ComN expression from the *comNOPQ* operon has therefore for many years been one of several USS-receptor candidates based on gene regulatory and null-mutant phenotypes, yet functional characterization of protein function and role has remained unexplored. We found further that the *ppdA/comN* gene ontology and chromosomal localization were conserved across most of the Pasteurellaceae, in the same gene order as *comNOPQ* (Supplementary Figure S14). The operon was generally found located upstream of and in the same orientation as the *recC* gene. The *recC* gene encoding the exoribonuclease V gamma subunit is involved in recombination-dependent DNA repair and in the final stages of transformation involving homologous recombination (Helm and Seifert, 2009). The co-localization of the *comNOPQ* operon and *recC* therefore seems functionally linked to transformation across Pasteurellaceae. Interestingly, this *comNOPQ* genomic location and operonic organization was also found to be the same as *ppdAB-ygdB-ppdC* in *E. coli* and other Enterobacteriaceae genera (Supplementary Figure S14). The *E. coli* operon has equivalently been shown to be regulated by Sxy, and *ppdA* has also been shown to be the most strongly expressed gene upon induction, exactly like in *H. influenzae* (Sinha et al., 2009; Jaskólska and Gerdes, 2015). *E. coli* has low transformation rates (10^−7^–10^−8^) and is generally not considered a competent species, although the competence and type IV pilus proteins are encoded in the genome (Riva et al., 2020; Claverys et al., 2009).

### PpdA_Past_ structural insights and future outlook

We reported the AlphaFold-predicted topology and 3D structure of PpdA_Past,_ showing its similarity to other characterized minor pilins with established roles in DNA-binding in support of a USS receptor candidacy. The conserved gene regulatory organization and strong structural coherence of PpdA across phylogenetic distances within the Pasteurellaceae family support one conserved function in transformation. Although the aligned amino acid identity between FimT*_Lpne_* and PpdA*_Hinf_* was only 15.5%, the atomic Cα positions of full-length PpdA*_Hinf_* were, on average, less than 1.3 atomic radii away (RMSD 1.208) from the aligned atoms of the here modeled full-length FimT*_Lpne_*. This single observation implies that PpdA_Past_ belongs to the same functional class as FimT*_Lpne_* of DNA-binding minor pilins. Identifying conserved electropositive/polar residues in PpdA_Past_ is involved in DNA-binding in the non-sequence specific FimT*_Lpne_* and the sequence specific ComP*_Neis_* corroborates this understanding. Based on these structural interpretations, we also deductively hypothesize that the homologous PpdA of *E. coli* and other Enterobacteriaceae have DNA-binding properties. Further experiments investigating the DNA-binding properties of PpdA*_Ecol_* could perhaps help explain if low affinity for DNA could be impacting the relatively low transformation rates in this important model organism. Although FimT*_Lpne_* and PpdA*_Hinf_* are structural homologs, they differ in their C-terminal domains, which potentially could explain differences in DNA-binding preferences. PpdA_Past_ was found to share with ComP*_Neis_* a β-sheet transversing loop anchored by a disulfide bridge, which in PpdA*_Past_* similarly may be involved in sequence specific DNA-binding. We and others have previously expected DUS and USS specificity to be an example of convergent evolution since the DUS/USS motifs are completely different, but identifying another minor pilin as the strongest USS-receptor candidate in Pasteurellaceae could rather suggest divergent evolution with deep roots in the Proteobacteria. Notably, a typical overall DNA-binding mode was found in top scoring PpdA_USS_ and PpdA_eUSS_ models of both *Hin-*USS and *Apl-*USS species suggesting one conserved and coherent DNA-binding mode. Furthermore, structural modeling of PpdA-homologs and DNA from different bacteria should be undertaken to determine if there is one unified binding mode. Finding that PAE of the PpdA_USS_ and PpdA_scr_ models on average were not significantly different from each other supports the existence of one robust binding mode. Since the USS is not palindromic, we could explore the orientation of the USS in the PpdA_USS_ models and find both in robust models. One of the USS orientations showed USS-dialect variable base pairs in proximity to PpdA_Past_. This modeled proximity was in the C-terminal region of PpdA with the β-sheet transversing loop resembling the DUS specific ComP as discussed above. It is therefore tempting to speculate that the two USS dialects, *Apl*- and *Hin*-USS, have diverged into their USS specificities from their common ancestry through mutations in the C-terminal part of PpdA. This evolutionary scenario aligns with the general phenomenon that the evolutionary rate is generally higher at protein termini (Bricout et al., 2023). An implication for this USS orientation model is that the inner core of USS, conserved in both dialects, would interact with domains closer to the N-terminal part of the protein toward the α-helix. The same USS orientation was supported in the molecular dynamics simulations in finding a lower median RMSD (1.46 Å) for this USS-oriented complex than the opposite (2.29 Å). The coevolution signals, however, are dispersed across PpdA and do not cluster in the C-terminal part, making this interpretation of USS-orientation warranting further investigation.

## Conclusion

PpdA of the Pasteurellaceae (PpdA_Past_) was predicted to be the USS-receptor by protein-DNA complex modeling in AlphaFold3. PpdA_Past_ was the only Pasteurellaceae orthogroup out of 73 to form significantly better models with USS than scrambled DNA. In contrast to the few other orthogroups which formed robust protein-DNA complexes, PpdA_Past_ was widely distributed across the Pasteurellaceae enriched in USS. Protein-DNA sequence specificity predictions using DeepPBS showed that USS was significantly predicted as a DNA-binding motif by PpdA_Past_. Of the 73 explored orthogroups, PpdA_Past_ had among the strongest coevolutionary signals with USS and particularly with USS-dialect-defining nucleotides. PpdA as minor pilin and USS-receptor was found to fit current models of transformation in sharing structural features with other minor pilin DNA-receptors and from existing knowledge on its gene regulation and null transformation phenotype of the *H. influenzae* knock out. Only one robust PpdA_Past_ DNA-binding mode was identified, yet with two opposite USS orientations. Coevolutionary signals in the PpdA_Past_ C-terminal β-sheet was identified which together with AF3 models and molecular dynamics simulations lent support to one of the two alternative USS orientations. We propose the name ComN to be used for PpdA_Past_.

## Data Availability

The data and code presented in this study are publicly available at: https://github.com/stianale/USS-receptor.

## Footnotes

^1^ https://github.com/stianale/USS-receptor

^2^ https://github.com/ParBLiSS/FastANI

^3^ https://inkscape.org/

^4^ https://bioinformatics.org/aces/miBio

^5^ alphafoldserver.com
