# Supplementary material for: Type IV minor pilin ComN predicted the USS-receptor in Pasteurellaceae
Source: Front Microbiol. 2025 Oct 31;16:1647523. doi: 10.3389/fmicb.2025.1647523 (PMC12616744; doi:10.3389/fmicb.2025.1647523)
Supplement: Supplementary file 1 [file Data_Sheet_1.docx]

**Supporting Information**

**Type IV minor pilin ComN predicted the USS-receptor in Pasteurellaceae**

Stian Aleksander Helsem^1^, Kristian Alfsnes^2^, Stephan A. Frye^3^, Alexander Hesselberg Løvestad^1,2^ and Ole Herman Ambur^1^*

^1^Department of Life Sciences and Health, Faculty of Health Sciences, OsloMet, Norway

^2^Division for Infection Control, Norwegian Institute of Public Health, 0213 Oslo, Norway

^3^Division of Laboratory Medicine, Dept. Microbiology, Oslo University Hospital, Norway

*Correspondence: [olam@oslomet.no](mailto:olam@oslomet.no)

No. of supporting Figures: 15

No. of supporting Videos: 2

No. of supporting Tables: 9

No. of supporting data: 1

No. of supporting Code output: 1

**Supporting results and discussion**

**DeepPBS DNA-binding specificity predictions for *Hin-*USS species**

Predictions of PpdA*_Hin_*_-USS_ models of *H. influenzae* Rd modeled with variable significance (* p<0.05, ** p<0.01 and *** p<0.001) overrepresented nucleotides matching *Hin-USS* in positions 1(A), 2(A), 3(G), 4(T), 5(G), 6(C), 7(G) and 8(G) (Fig. S4 A; Table S4). Position 9(T) was non-significantly different from random, yet on the overrepresented side together with the significantly (***) overrepresented pyr-pyr transition 9(C). Both permutational transversions 9(G) and 9(A) were significantly (***) underrepresented. The descending order of mean predicted probabilities for each *Hin-*USS nucleotide was 4(T), 6(C), 8(G), 2(A), 7(G), 1(A), 5(G), 3(G) and 9(T). The sequence logo of *Hin-*USS from this species showed that the weakest predicted 9(T) also to be the least conserved nucleotide relative to the other USS positions and the significantly (***) underrepresented 9(G) to be the rarest permutation (Fig. S4 A)

AF3 models of PpdA*_Hin-_*_USS_ models of *Mannheimia succiniciproducens* strain MBEL55E predicted with variable significance (*to ***) overrepresented nucleotides matching *Hin-*USS in all positions 1-9 (Fig. S4 B; Table S4). Positions 3(G) and 5(G) had weaker overrepresented significance (* and **) than the other six nucleotide positions (***). The descending order of mean predicted probabilities for each *Hin-*USS nucleotide was 1(A), 4(T), 6(C), 9(T), 2(A), 7(G), 8(G), 5(G) and 3(G). The sequence logo of the USS for this species showed that the weakest predicted position 3(G) to also be markedly less conserved relative to all other *Hin-*USS positions and notably relative to the other *Hin-*USS species. (Fig. S4 B)

AF3 models of PpdA*_Hin-_*_USS_ of *A. actinomycetemcomitans* strain 31S predicted with strong significance (***) overrepresented nucleotides matching *Hin-*USS in position 1(A), 2(A), 4(T) and 8(G) (Fig. S4 C; Table S4). In contrast, position 3(G) to match *Hin-*USS was significantly (**) underrepresented and the complementary pur-pyr permutational transversion 3(C) was significantly (***) overrepresented. Positions 5(G), 6(C) and 7(G) were non-significantly different from random and the complementary permutational transversions 5(C), 6(G) and 7(C) were significantly (** and ***) underrepresented. The descending order of mean predicted probabilities for each USS nucleotide was 4(T), 8(G), 1(A), 2(A), 5(G), 7(G), 6(C), 9(T) and 3(G). The sequence logo of the *Hin-*USS in this species showed that the weakest and predicted underrepresented position 3(G) was also among the least conserved of the *Hin-*USS positions (Fig. S4 C).

AF3 models of of PpdA*_Hin-_*_USS_ of *Aggregatibacter sp.* oral taxon 513 predicted with variable significance (* to ***) overrepresented nucleotides matching *Hin-*USS in positions 1(A), 2(A), 3(G), 4(T), 7(G), 8(G) and 9(T) (Fig. S4 D; Table S4). Positions 5(G) and 6(C) were non-significantly different from random, yet on the overrepresented side together with their significantly overrepresented permutational transitions 5(A)* and 6(T)***, respectively. The descending order of mean predicted probabilities for each *Hin-*USS nucleotide was 4(T), 7(G), 8(G), 1(A), 3(G), 2(A), 9(T), 6(C) and 5(G). Although non-significantly predicted by DeepPBS, positions 5(G) and 6(C) are among the best conserved nucleotides relative to the other USS positions in the USS sequence logo in this species (Fig. S4 D).

AF3 models of PpdA*_Hin_*_-USS_ models of *P. multocida* strain NCTC8282 predicted with strong significance (***) overrepresented nucleotides matching *Hin-*USS in positions 1(A), 3(G), 4(T) and 9(T) (Fig. S4 E; Table S4). All other positions 2(A), 5(G), 6(C), 7(G) and 8(G) were non-significantly different from random. The descending order of mean predicted probabilities for each *Hin-*USS position was 4(T), 3(G), 9(T), 1(A), 6(C), 2(A), 5(G), 8(G) and 7(G). In the sequence logo, the weakest predicted position 7(G) was also the least conserved relative to the other *Hin-*USS positions and in this species also relative to the other *Hin-*USS species. Also, the *P. multocida* NCTC8282 USS differed from the other *Hin-*USS sequence logos by having a relatively less conserved 6(C) and a particularly conserved 9(T) which was uniquely better conserved than 7(G) and 8(G) in this species. It is notable that the weakest predicted and least conserved position in *H. inf* Rd 9(T) was both predicted with strong significance and was better conserved in *P. multocida* (Fig. S4 E).

Considering similarities in the predictions for each species (Fig. S4 A-E, *H. inf* Rd PpdA*_Hin_*_-USS_ and *A. sp.* oral taxon 513 PpdA*_Hin_*_-USS_ were most similarly predicted across *Hin-*USS with particularly strong predictions for 4(T), 6(C), 7(G) and 8(G), also similar to *M. succiniciproducens* strain MBEL55E with a strong additional 1(A) prediction.

**DeepPBS DNA-binding specificity predictions for *Apl-*USS species**

AF3 models of PpdA*_Apl_*_-USS_ of *Actinobacillus equuli* subsp. haemolyticus strain 3524 predicted with strong significance (***) overrepresented nucleotides matching *Apl-*USS in positions 1(A), 2(C), 3(A), 4(A), 6(C), 7(G), 8(G) (Fig. S5 A, Table S4). Position 5(G) to match *Apl-*USS was underrepresented with weaker significance (*) below random chance (25%) and the non-complementary transversion permutation 5(T) was overrepresented with strong significance (***). Position 9(T) to match *Apl-*USS was underrepresented with strong significance (***) and the non-complementary transversion permutation 8(G) was overrepresented (***). The descending order of mean predicted probabilities for each *Apl-*USS nucleotide was 1(A), 3(A), 7(G), 8(G), 2(C), 4(A), 6(C), 5(G) and 9(T). The USS sequence logo from this species showed that the significantly overrepresented positions 3(A), 4(A), 6(C), 7(G) were also the most conserved nucleotides relative to the other USS positions. In contrast, positions 1(A) and 2(C) were the least conserved, yet significantly overrepresented (***). Underrepresented position 5(G) was less conserved than the adjacent 3(A), 4(A), 6(C) and 7(G) pairs relative to the other *Apl-*USS species except *Actinobacillus lignieresii* strain NCTC4189 with a resembling sequence logo (Fig. S5 A-E).

AF3 models of PpdA*_Apl_*_-USS_ of *Frederiksenia canicola* strain HPA 21 predicted with strong significance (***) overrepresented nucleotides matching *Apl-*USS in positions 1(A), 2(C), 3(A), 4(A), 6(C), 7(G) and 8(G) (Fig. S5 B, Table S4). Positions 5(G) and 9(T) were underrepresented with strong significance (***). In position 5(G) both the complementary transversion permutation 5(C) and the transition permutation 5(A) were significantly (***) overrepresented. In position 9(T) the non-complementary transversion permutation 9(G) was significantly (***) overrepresented. The descending order of mean predicted probabilities for each *Apl-*USS nucleotide was 8(G), 7(G), 1(A), 2(C), 4(A), 3(A), 6(C), 9(T) and 5(G). The USS sequence logo from this species showed that the significantly overrepresented positions 3(A), 4(A), 6(C), 7(G), 8(G) were also the most conserved nucleotides relative to the other USS positions and together with the conserved yet underrepresented 5(G). 1(A), 2(C) and 9(T) were less conserved than the above and 1(A) the least (Fig. S5 A-E).

AF3 models of PpdA*_Apl_*_-USS_ models of Pasteurellaceae bacterium Orientalotternb1 predicted with strong significance (***) overrepresented nucleotides matching *Apl-*USS in positions 2(C), 3(A), 4(A), 7(G) and 8(G) (Fig. S5 C, Table S4). Position 1(A) was underrepresented with weak significance (*) and the position significantly overrepresented with the non-complementary transversion permutation 1(C)* and the transition permutation 1(G)***. Position 6(C) was underrepresented with strong significance (***) and the position significantly overrepresented with the transition permutation 6(T)***. Positions 5(G) and 9(T) were non-significantly different from random. The descending order of mean predicted probabilities for each *Apl-*USS nucleotide was 2(C), 3(A), 4(A), 8(G), 7(G), 6(C), 9(T), 5(G) and 1(A). The USS sequence logo from this species showed that the weakest predicted and underrepresented 1(A) was also the least conserved. In contrast, the strongest predicted 2(C) was less conserved than positions 3-9 (Fig. S5 A-E).

AF3 models of PpdA*_Apl_*_-USS_ models of *Mannheimia bovis* strain 39324S-11 predicted with strong significance (***) overrepresented nucleotides matching *Apl-*USS in positions 2(C), 3(A), 4(A), 7(G) and 9(T) (Fig. S5 D, Table S4). Positions 1(A) and 5(G) were underrepresented with strong significance (***). In position 1(A) the transition permutation 1(G) was significantly (***) overrepresented. In positions 5(G) both the complementary transversion permutation 6(T) and the transition permutation 6(A) were significantly (**) overrepresented. 6(C) and 8(G) were non-significantly different from random and both positions significantly overrepresented with their respective transition permutations 6(T) and 8(A), respectively. The descending order of mean predicted probabilities for each *Apl-*USS nucleotide was 2(C), 3(A), 4(A), 9(T), 7(G), 6(C), 8(G), 5(G) and 1(A). The USS sequence logo from this species showed that both the weakest predicted 1(A) and the strongest predicted 2(C) were less conserved than positions 3-8 and with 1(A) the least. A unique feature of this species’ sequence logo was that position 7(G) and 9(T) were equally conserved where the other *Apl-*USS sequence logos showed better conservation for 7(G) than 9(T) (Fig. S5 A-E).

AF3 models of PpdA*_Apl_*_-USS_ models of *Actinobacillus lignieresii* strain NCTC4189 predicted with strong significance (***) overrepresented nucleotides matching *Apl-*USS in positions 1(A), 2(C), 3(A), 4(A), 6(C) and 9(T) and less significantly positions 7(G)** and 8(G)* (Fig. S5 E, Table S4). Position 5(G) to match *Apl-*USS was non-significantly different from random and no other nucleotide was overrepresented in this position. The descending order of mean predicted probabilities for each *Apl-*USS nucleotide was 3(A), 2(C), 6(C), 4(A), 1(A), 7(G), 9(T), 8(G) and 5(G). The USS sequence logo from this species resembled that of *A. equuli* subsp. haemolyticus strain 3524 with a less conserved 5(G) relative to the 3(A), 4(A), 6(C) and 7(G), equally conserved 8(G) and 9(T) and less conserved 2(C) relative to the other *Apl-*USS sequence logos. The least conserved nucleotide position was also in this species 1(A) with a strong prediction (Fig. S5 A-E).

Overall, DeepPBS predicted the near exact overrepresented nucleotides matching *Apl-*USS with variable significance (Fig. S6; Table S4; Video S2). *Apl*-USS positions 1(A), 2(C), 3(A), 4(A), 6(C), 7(G) and 8(G) were predicted overrepresented with strong significance (***). Notably, positions 2-4 which distinguish *Apl-*USS from *Hin-*USS and 2(C) were strongly predicted whilst having generally lower levels of conservation across *Apl-*USS species. Position 9(T) was predicted overrepresented with weak significance (*) together with the significantly (***) overrepresented non-complementary transversion permutation 9(G). Position 5(G) was found significantly underrepresented (***) and the non-complementary transversion permutation 5(T) significantly (***) overrepresented. The descending order of mean predicted probabilities was 2(C), 3(A), 4(A), 6(G), 7(G), 1(A), 6(C), 9(T) and 5(G). The only predicted underrepresented position 5(G) was found relatively less conserved in the USS sequence logos of two species (*A. equuli* subsp. haemolyticus strain 3524 and *A. lignieresii* strain NCTC4189 ). Although 1(A) was the consistently least conserved it was predicted significantly overrepresented (***) in the PpdA*_Apl_*_-USS_. In considering the ambiguity of the two highest mean predicted probability nucleotides of each *Apl-*USS position, position 1, 2, 3, 6, and 8 were all transition permutations (Pyr-Pyr and Pur-Pur), whereas position 4(A) was alternatively predicted with the complementary transversion 4(T) and position 9(T) with the non-complementary transversion permutation 9(G). It is noteworthy that DeepPBS efficiently distinguished the USS dialect-specific nucleotides (AGT/CAA) yet predicts the GCGG-core common to both *Hin-* and *Apl-*USS less convincingly. This is exemplified by the high mean probabilities for *Hin*-USS AGT and *Apl*-USS CAA in positions 2-4 for PpdA*_Hin-_*_USS_ and PpdA*_Apl_*_-USS_, respectively, as well as the strong prediction of **TT**GG in place of the consensus inner **GC**GG-core for PpdA*_Apl_*_-USS_ in terms of mean probability. It could be that there are elements of the DNA-binding mode of PpdA*_Hin-_*_USS_ AF3 models that accounts for this discrepancy/peculiarity. We include two videos (Supporting Videos 1 and 2) showing how the DeepPBS predictions change with the dynamically flexing PpdA-USS complex where individual frames will be analyzed for co-varying dynamic amplitudes in the nucleotide predictions in subsequent work.

**Coevolution analysis of PpdA with Cramér’s Φ correlations to eUSS**

The PpdA correlations to each eUSS position showed that the first eUSS position 1(A/T) was correlated with many positions throughout PpdA, having the strongest correlations with PpdA positions 91, 149 and 68 (Φ*c* = 0.939; Φ*c*= 0.927; Φ*c*=0.894) (Table S7; Fig. S8). eUSS Position 2(A) did not vary across sequences and thus had no significant pairwise correlations with PpdA positions. All three eUSS positions 3(A/C), 4(G/A) and 5 (T/A) were correlated with the same positions in PpdA and of equal magnitude. These three positions distinguish the 9-mer *Hin-*USS [3(A),4(G) and 5(T)) from *Apl-*USS (3(C), 4(A) and 5(A)]. Since these three positions also separate the *Hin-*USS and *Apl-*USS phylogenetic clades, the heatmap highlights that many of the amino acids across PpdA are co-evolved (drift and selection) with each specificity. Several very strong PpdA correlations (Φ*c* = 1) were found with these three eUSS positions, for example positions 12, 116 and 174 in PpdA (Table S7). The 9-mer inner-core GCGG (eUSS positions 6(G), 7(C), 8(G) and 9(G)) and eUSS position 10(T) were invariable, as shown by the blue columns in Fig. S8. eUSS position 11(C/T/G/A) contained all four nucleotides, with C the most prevalently predicted, and was correlated with several PpdA positions (Table S7). The strongest correlations were with respective PpdA positions 187 (Φ*c*=0.850), 133 (Φ*c*=0.834) and 191 (Φ*c*=0.812). eUSS position 12(A/T/G) was predicted predominantly A, less T and G, and had moderately high correlations with PpdA positions 48 (Φ*c*=0.702); 50 (Φ*c*=0.603) and 98 (Φ*c*=0.598) and low to moderate correlations with other positions (Table S7). eUSS position 13 (A/G/T) was predicted with A, less G and T, and had several correlations with PpdA positions, of which 50 (Φ*c*=0.801), 71 (Φ*c*=0.786) and 147 (Φ*c*=0.738) were the strongest. eUSS position 14(A/T) was predicted near equal amounts of A and T and was strongly associated with the three PpdA positions 153 (Φ*c*=0.796), neighboring 152 (Φ*c*=0.789) and 125 (Φ*c*=0.773). eUSS positions 15(T) and 16(T) were invariable as shown by the blue columns in Fig. S8. eUSS position 17(T/C) was predicted with mostly T and a small number of C and were strongly correlated (Φ*c*=1) with the three PpdA positions 42, 147 and 170 (Table S7).

For eUSS position 1, two of the top three PpdA positions in the Φ*c* analysis were also among the top three correlating positions in the miBIO analysis, with the highest MI values being PpdA positions 25, 91 and 149, respectively (Tables S8 and Fig. 5). The invariable eUSS position 2(A) correlated with exactly three PpdA positions (16, 198 and 12) with positive MI values, albeit weakly positive. The USS dialect specific eUSS positions 3-5 correlated with PpdA position 11 with highest MI being the same position that ranked on top in Φ*c*, while multiple sites ranked second in both miBIO and Φ*c* such as PpdA position 116. In contrast to the Φ*c* analysis, miBIO results showed eUSS positions 6-10 to weakly correlate with different PpdA positions of which 7 and 16 stood out. eUSS position 11 had PpdA positions 191, 133 and 98 as the three with the highest MI, of which the two former were also among the top ranking three in Φ*c*. For eUSS position 12, PpdA positions 89, 47 and 117 ranked among the top three, respectively, while for eUSS position 13, PpdA positions 177, 175 and 71 ranked top three. eUSS position 14 had PpdA positions 151, 97 and 122 ranked the top three, while eUSS position 15 correlated with PpdA positions 7, 16 and 11 as the top three. For eUSS position 16, PpdA positions 7, 16 and 198 ranked the top three. eUSS position 17 correlated with several PpdA positions of which 42, 147 and 170 ranked first and among top three in the miBIO and Φ*c* analysis, respectively.

**Supplementary information legends and statistical analysis**

**Video S1.** DeepPBS predicted DNA-binding specificity for *H. inf* Rd 98/200 PpdA_Hin-USS_ AF3 models with ipTM > 0.6 (number of frames/models) concatenated into GIF format. Upper left panel: AF3 input Hin-USS sequence. Bottom left panel: DeepPBS prediction. Right panel: Sequence logos showing the relative proportions of the nucleotides at each position. Position 0 refers to USS position 1.

**Video S2.** DeepPBS predicted DNA-binding specificity for 98/200 *Actinobacillus equuli* PpdA_Apl-USS_  AF3 models with ipTM > 0.6 concatenated into GIF format. Upper left panel: AF3 input Apl-USS sequence. Bottom left panel: DeepPBS prediction. Right panel: Sequence logos showing the relative proportions of the nucleotides at each position.

**Supplementary Data 1**
PpdA_Past_ alignment across all 150 OD species generated using MView v. 1.68 [(1)](https://www.zotero.org/google-docs/?Ra9W4M)

**Supplementary Code Output S1**

Wilcoxon rank sum tests on ipTM, PAE, CPPM and pLDDT (orthogroup_USS_ vs. orthogroup_scr_).

> results <- new_df %>%

+ group_by(Protein_HOG) %>%

+ filter(n_distinct(Scrambled) > 1) %>% # Ensure both "Yes" and "No" are present

+ summarise(p_value = list(wilcox.test(ipTM ~ Scrambled)$p.value), .groups = "drop") %>%

+ unnest(p_value)

Warning message:

There was 1 warning in `summarise()`.

ℹ In argument: `p_value = list(wilcox.test(ipTM ~ Scrambled)$p.value)`.

ℹ In group 2: `Protein_HOG = "hypothetical protein | N0.HOG0002116"`.

Caused by warning in `wilcox.test.default()`:

! cannot compute exact p-value with ties

>

> print(results)

# A tibble: 3 × 2

Protein_HOG p_value

<chr> <dbl>

1 DUF4198 domain-containing protein | N0.HOG0001749 **0.0000156**

2 hypothetical protein | N0.HOG0002116 **0.178**

3 type II secretion system protein | N0.HOG0001148 **0.000000243**

>

> results <- new_df %>%

+ group_by(Protein_HOG) %>%

+ filter(n_distinct(Scrambled) > 1) %>% # Ensure both "Yes" and "No" are present

+ summarise(p_value = list(wilcox.test(PAE ~ Scrambled)$p.value), .groups = "drop") %>%

+ unnest(p_value)

>

> print(results)

# A tibble: 3 × 2

Protein_HOG p_value

<chr> <dbl>

1 DUF4198 domain-containing protein | N0.HOG0001749 **0.000226**

2 hypothetical protein | N0.HOG0002116 **0.0392**

3 type II secretion system protein | N0.HOG0001148 **0.808**

>

> results <- new_df %>%

+ group_by(Protein_HOG) %>%

+ filter(n_distinct(Scrambled) > 1) %>% # Ensure both "Yes" and "No" are present

+ summarise(p_value = list(wilcox.test(CPPM ~ Scrambled)$p.value), .groups = "drop") %>%

+ unnest(p_value)

Warning message:

There was 1 warning in `summarise()`.

ℹ In argument: `p_value = list(wilcox.test(CPPM ~ Scrambled)$p.value)`.

ℹ In group 2: `Protein_HOG = "hypothetical protein | N0.HOG0002116"`.

Caused by warning in `wilcox.test.default()`:

! cannot compute exact p-value with ties

>

> print(results)

# A tibble: 3 × 2

Protein_HOG p_value

<chr> <dbl>

1 DUF4198 domain-containing protein | N0.HOG0001749 **0.00011**9

2 hypothetical protein | N0.HOG0002116 **0.190**

3 type II secretion system protein | N0.HOG0001148 **0.0107**

>

> results <- new_df %>%

+ group_by(Protein_HOG) %>%

+ filter(n_distinct(Scrambled) > 1) %>% # Ensure both "Yes" and "No" are present

+ summarise(p_value = list(wilcox.test(pLDDT ~ Scrambled)$p.value), .groups = "drop") %>%

+ unnest(p_value)

>

> print(results)

# A tibble: 3 × 2

Protein_HOG p_value

<chr> <dbl>

1 DUF4198 domain-containing protein | N0.HOG0001749 **0.0000000551**

2 hypothetical protein | N0.HOG0002116 **0.0392**

3 type II secretion system protein | N0.HOG0001148 **0.0000636**

**References**

1. Brown, N. P., Leroy, C., & Sander, C. (1998). MView: a web-compatible database search or multiple alignment viewer. *Bioinformatics (Oxford, England)*, *14*(4), 380-381.
2. Dehal, P. S., Joachimiak, M. P., Price, M. N., Bates, J. T., Baumohl, J. K., Chivian, D., ... & Arkin, A. P. (2010). MicrobesOnline: an integrated portal for comparative and functional genomics. *Nucleic acids research*, *38*(suppl_1), D396-D400.
