# Supplementary figures and images for: Type IV minor pilin ComN predicted the USS-receptor in Pasteurellaceae
Source: Front Microbiol. 2025 Oct 31;16:1647523. doi: 10.3389/fmicb.2025.1647523 (PMC12616744; doi:10.3389/fmicb.2025.1647523)

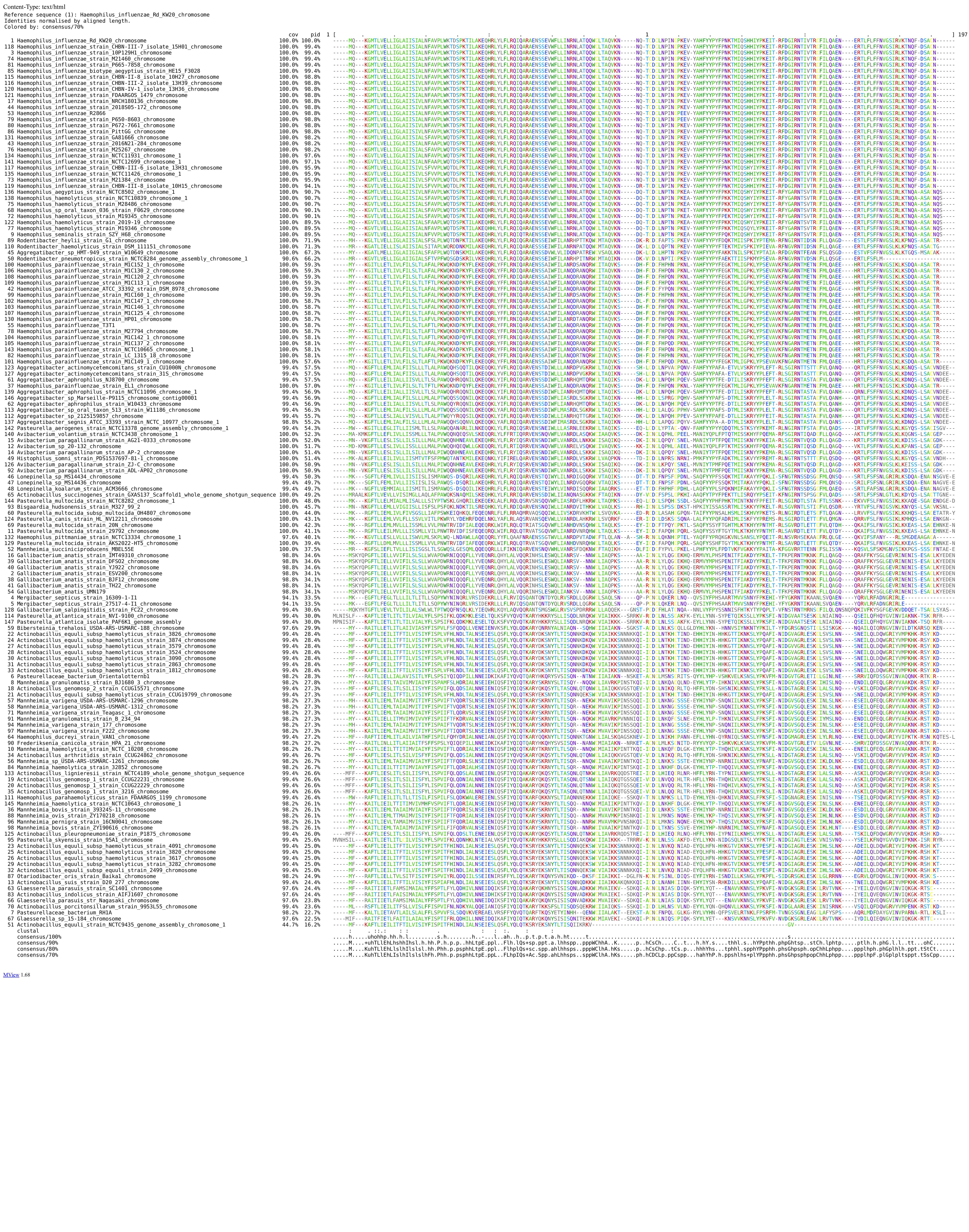

Supplement: Supplementary file 2 [file Image_1.jpeg]
