# Supplementary material for: Type IV minor pilin ComN predicted the USS-receptor in Pasteurellaceae
Source: Front Microbiol. 2025 Oct 31;16:1647523. doi: 10.3389/fmicb.2025.1647523 (PMC12616744; doi:10.3389/fmicb.2025.1647523)
Supplement: Supplementary file 3 [file Presentation_1.pdf]

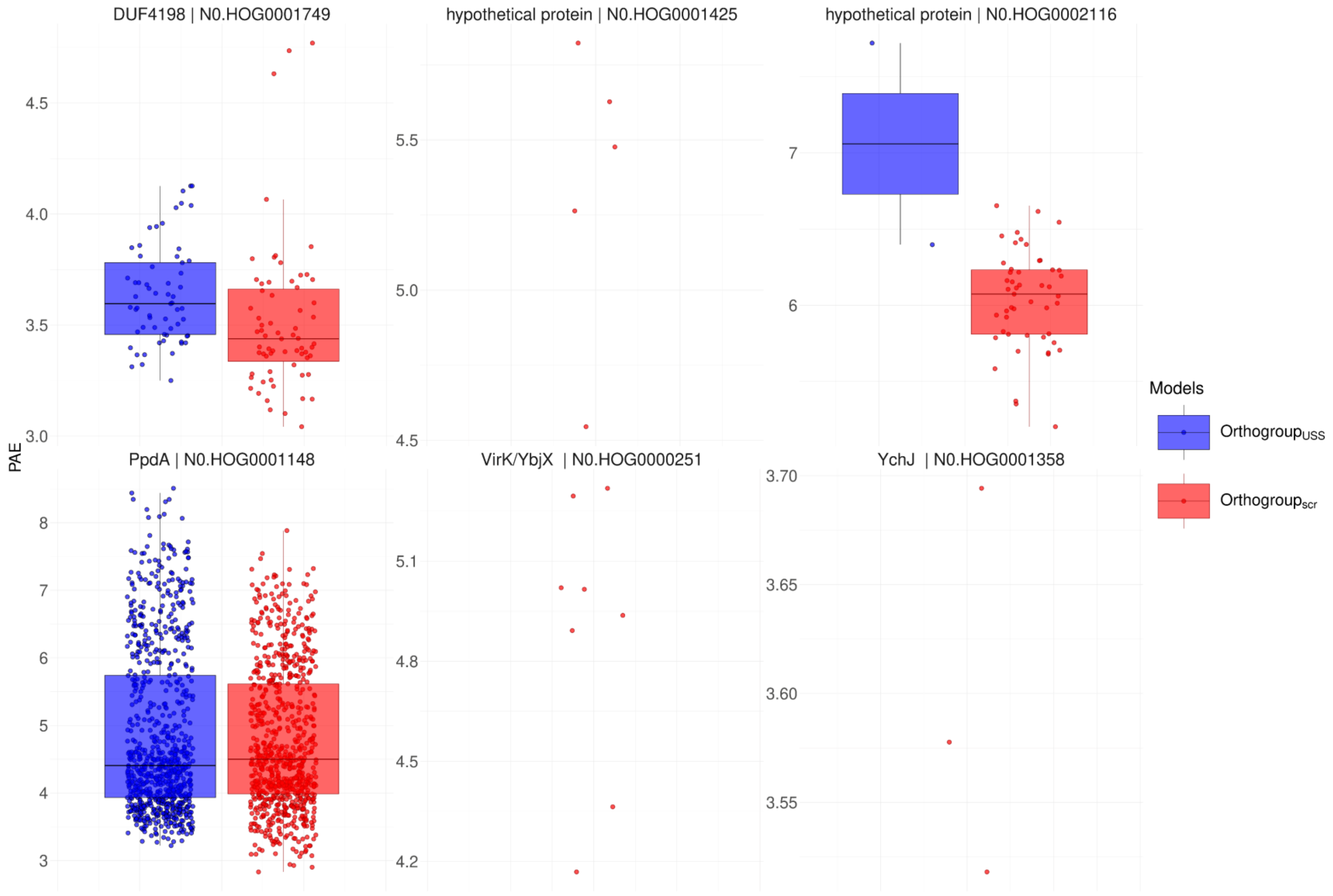

Figure S1. Box plots of distribution of PAE across all AF3 orthogroup$_{USS}$ and orthogroup$_{scr}$ models within ipTM$_r$ for the six modeled candidate proteins.

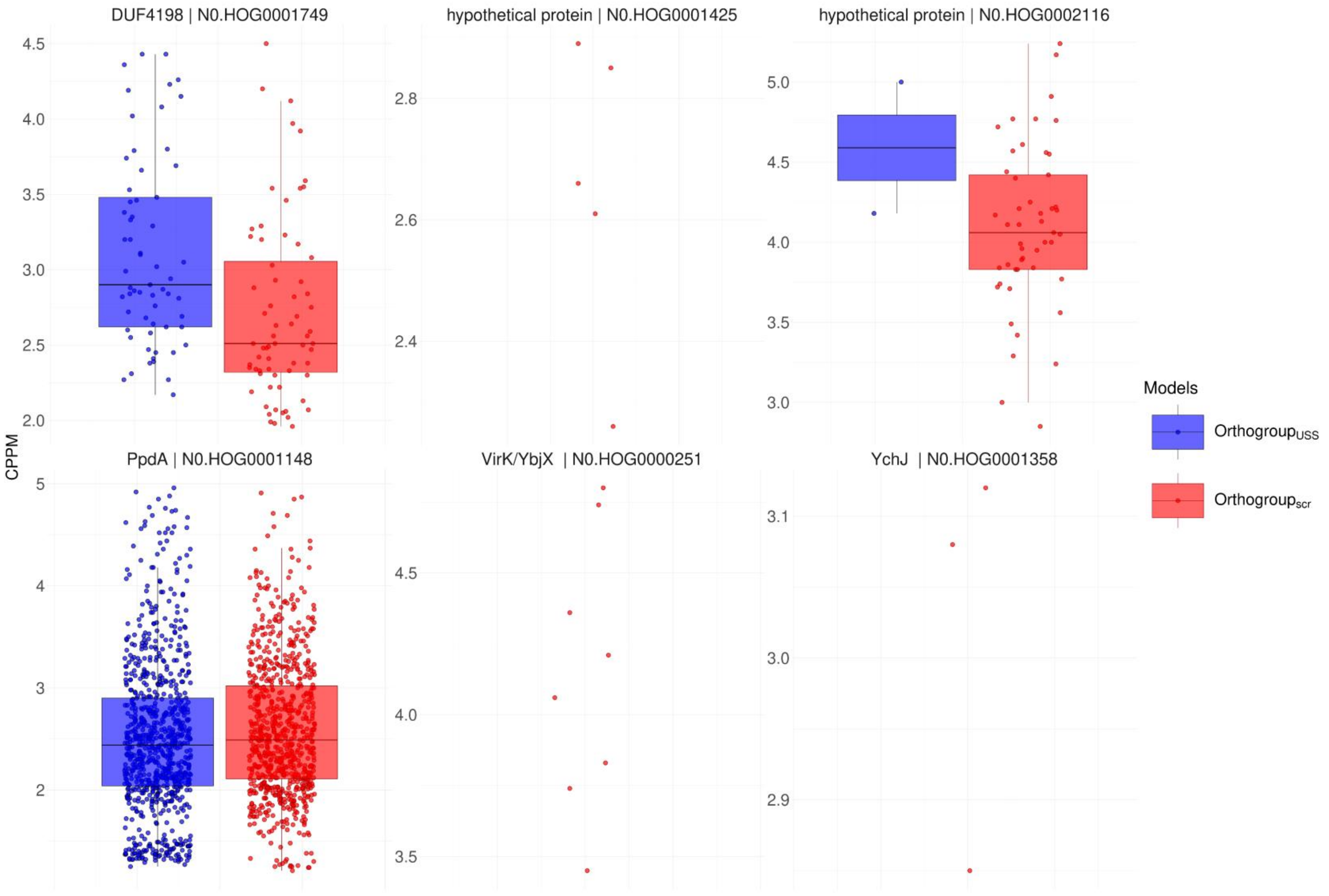

Figure S2. Box plots of distribution of CPPM across all AF3 orthogroup$_{USS}$ and orthogroup$_{scr}$ models within ipTM$_r$ for the six modeled candidate proteins.

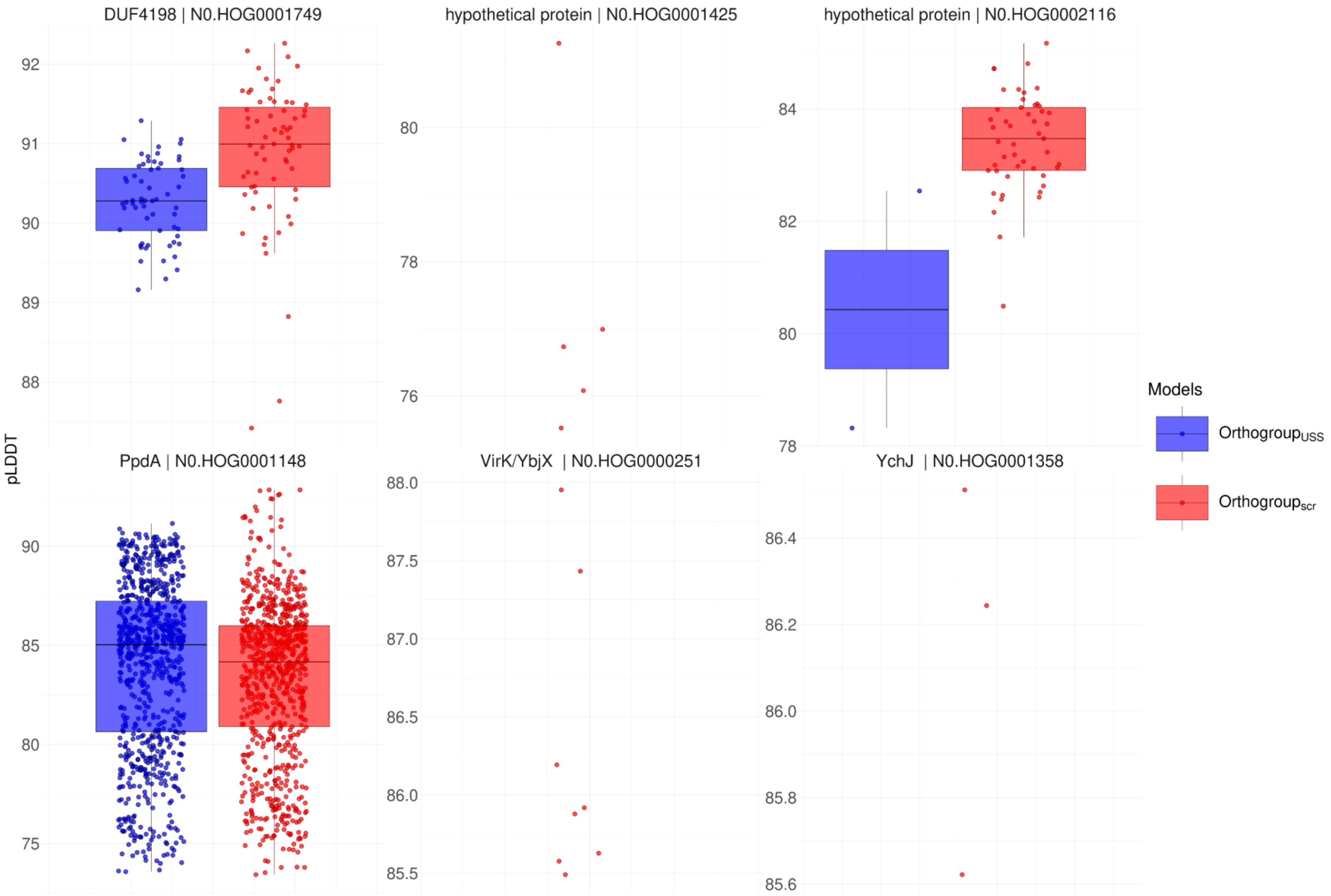

Figure S3. Box plots of distribution of pLDDT across all AF3 $\text{orthogroup}_{\text{USS}}$ and $\text{orthogroup}_{\text{scr}}$ models within ipTM_{r} for the six modeled candidate proteins.

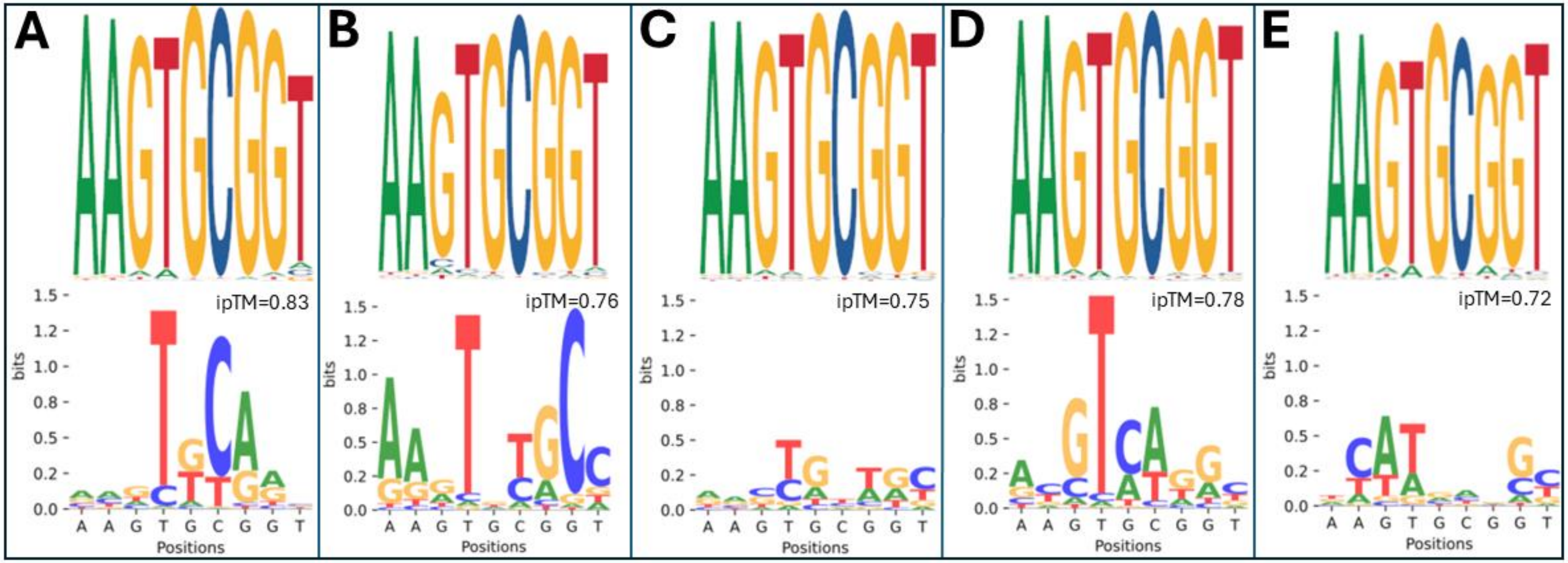

Figure S4. *Hin*-USS conservation and DeepPBS predictions.

Sequence logos of genome specific *Hin*-USS conservation (top panels) and mean predicted nucleotide frequencies in bits score from DeepPBS predictions on top-scoring (max ipTM) $\text{PpdA}_{\textit{Hin}\text{-USS}}$ AF3 inputs (lower panels). ipTM values for each of the AF3 input models are shown. **A:** *H. influenzae* Rd. **B**. *Mannheimia succiniciproducens* strain MBEL55E. **C**: *Aggregatibacter actinomycetemcomitans* strain 31S. **D**: *Aggregatibacter* sp oral taxon 513. **E**: *Pasteurella multocida* strain NCTC8282.

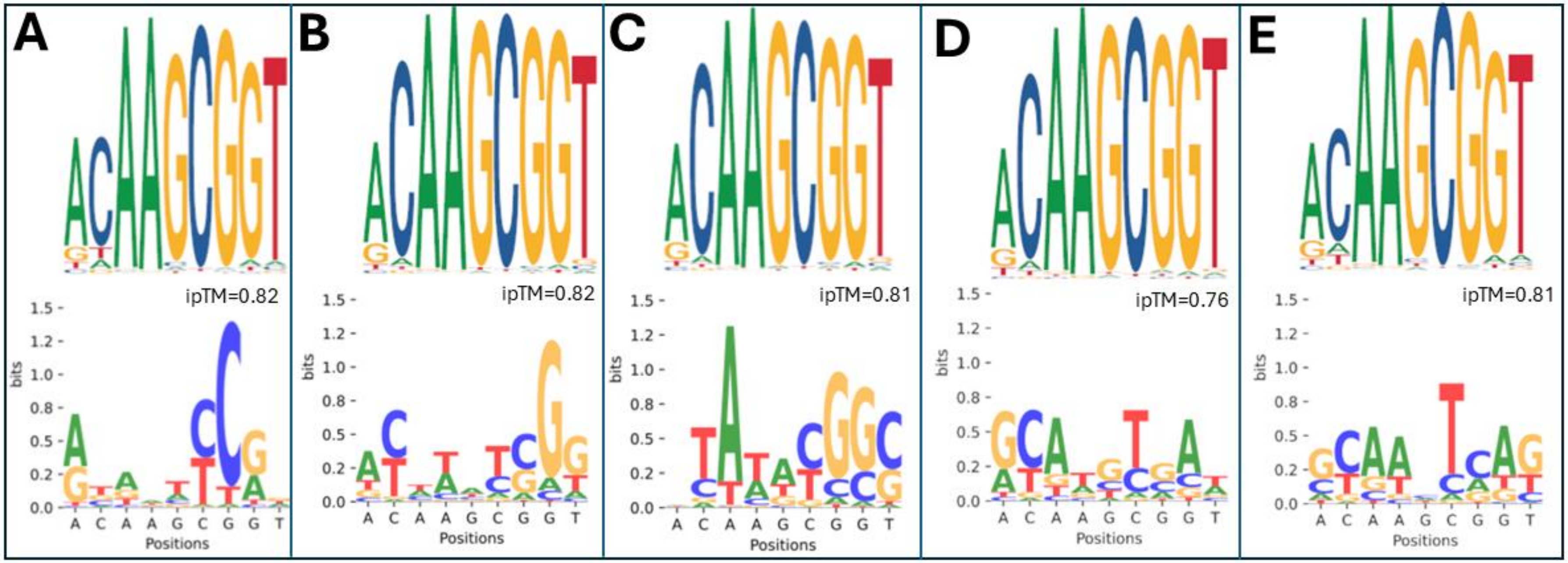

Figure S5. ***Apl*-USS conservation and DeepPBS predictions.**
Sequence logos of genome specific *Apl*-USS conservation (top panels) and mean predicted nucleotide frequencies in bits score from DeepPBS predictions on top-scoring (max ipTM) $PpdA_{Apl\text{-}USS}$ AF3 inputs (lower panels). ipTM values for each of the AF3 input models are shown. **A**: *Actinobacillus equuli subsp.* haemolyticus strain 3524. **B**: *Frederiksenia canicola* strain HPA 21. **C**: Pasteurellaceae bacterium Orientalotternb1. **D**: *Mannheimia bovis* strain 39324S-11. **E**: *Actinobacillus lignieresii* strain NCTC4189.

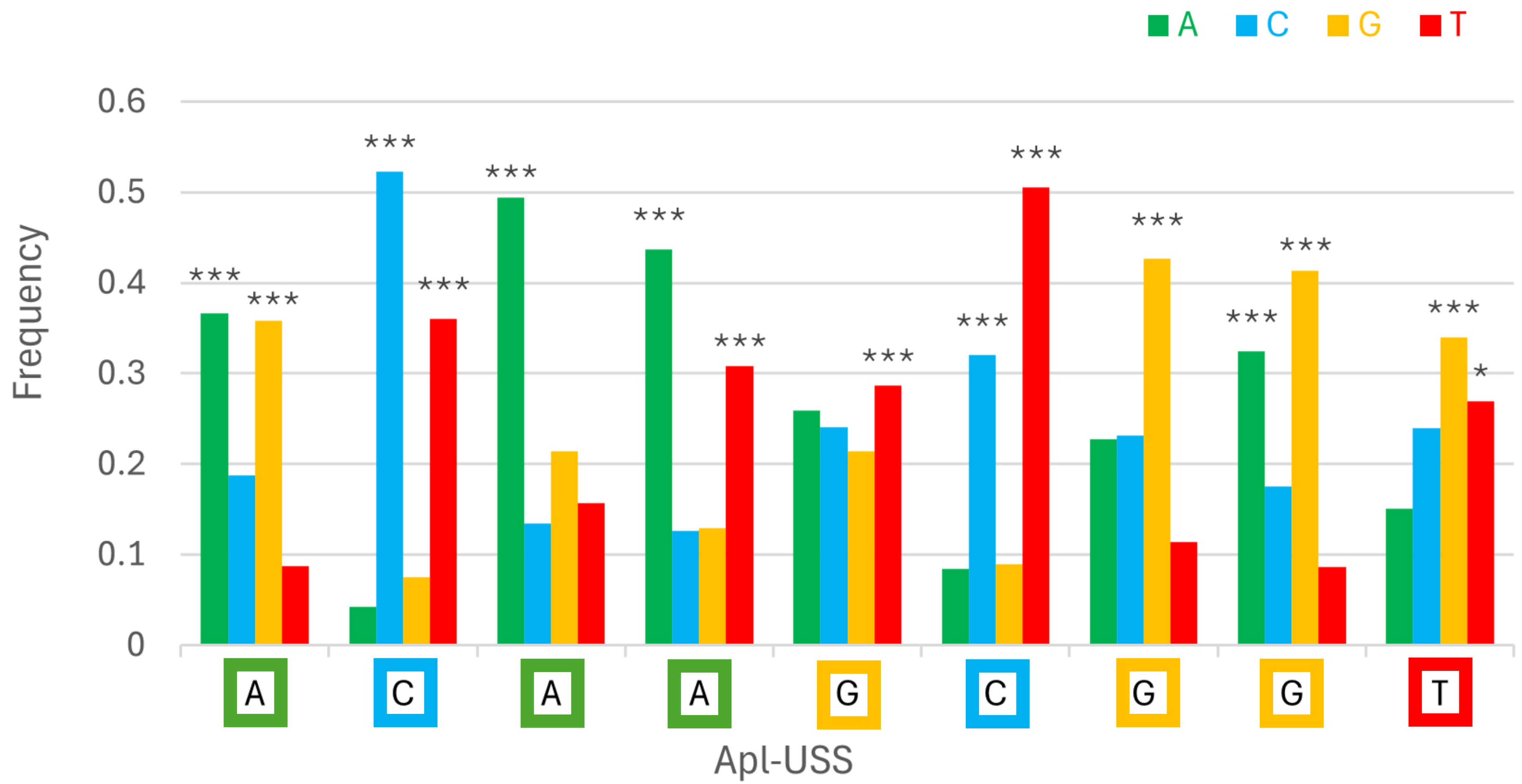

Figure S6. Consensus DeepPBS predictions of PpdA specificity against the reference *Apl*-USS. Distribution of consensus predicted nucleotide probabilities in each of the nine *Apl*-USS positions. Asterisks denote significant representation above random (0.25), ***P<0.001, **P<0.01, *P<0.05.

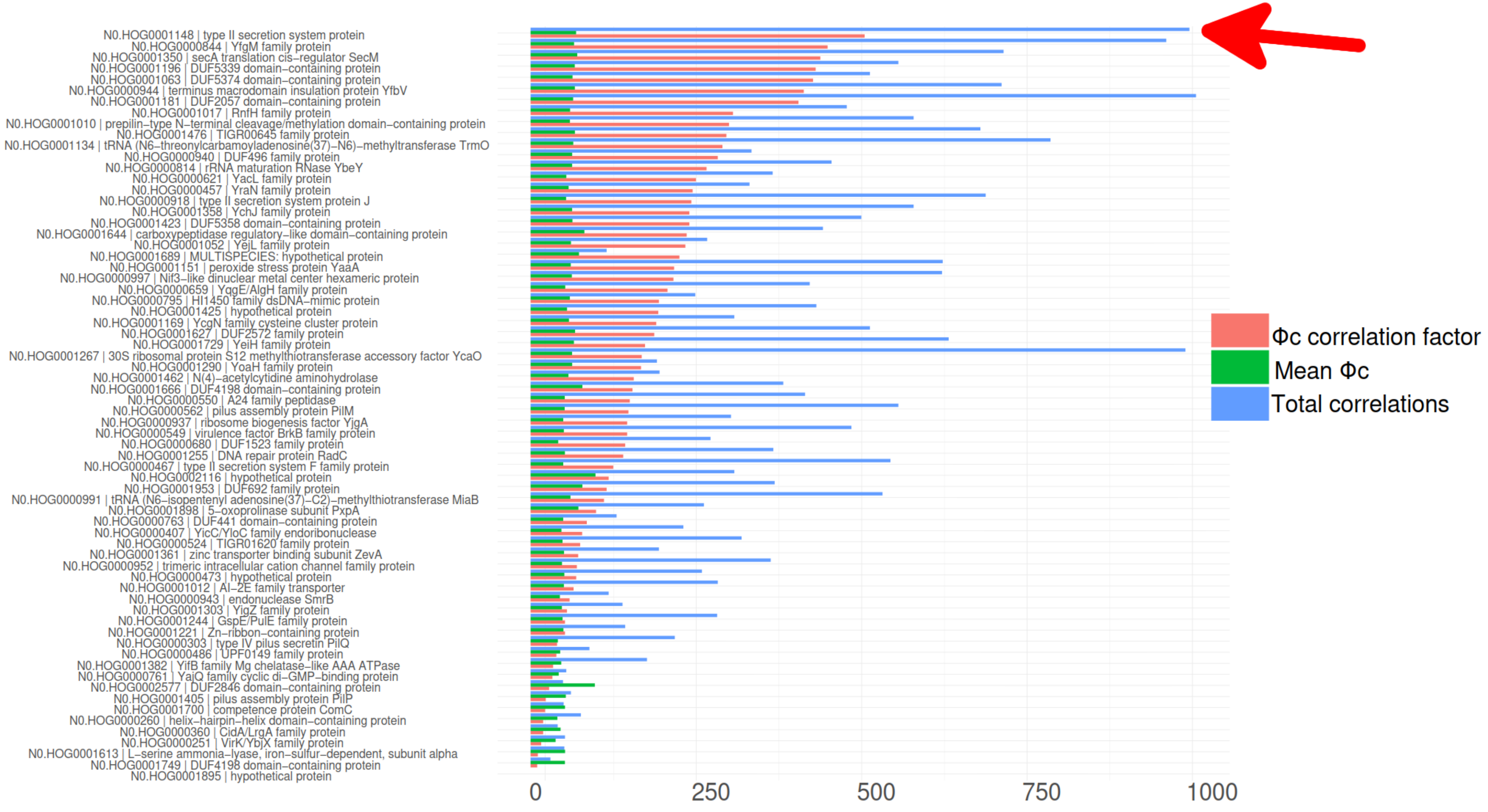

Figure S7. Coevolution of all modeled orthogroups and eUSS by Cramér's Φ.

Combined bar plots showing Φ*c* factor (red), mean Φ*c* (green) and total number of significant Φ*c* correlations (blue) for all orthogroups, sorted by mean Φ*c*. Φ*c* correlation factor and mean Φ*c* were multiplied by 100 to better scale with the number of Φ*c* correlations. Vertical axis labels: OrthoFinder hierarchical orthogroups and its most common gene name. Horizontal axis labels: Natural logarithm (ln). The relative positioning of PpdA = N0.HOG0001148 type II secretion system protein is indicated by red arrow.

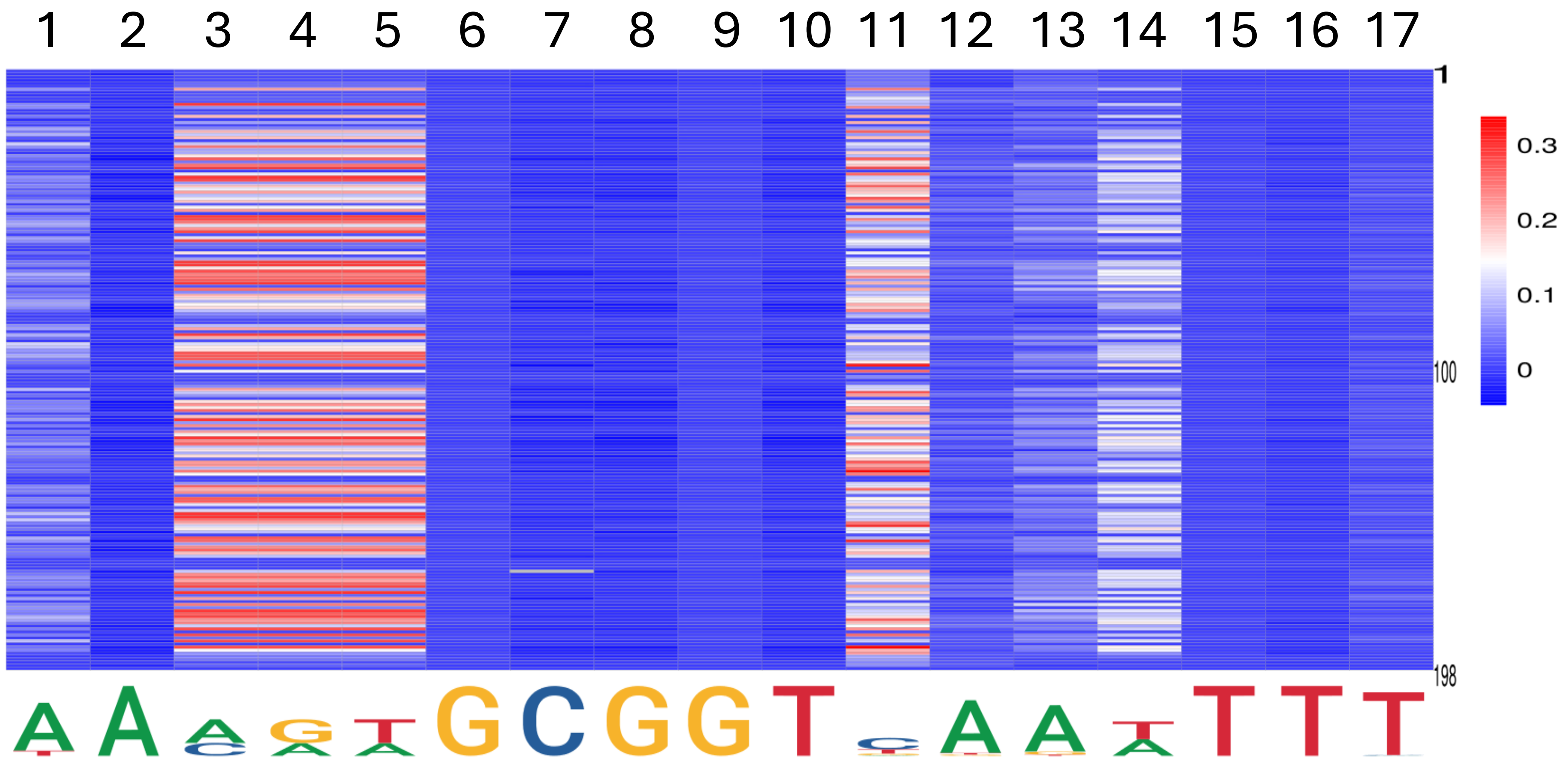

Figure S8. Graphical representation of the significant pairwise correlations for PpdA positions and eUSS by Cramér's Φ.
PpdA alignment positions on the vertical axis (1-198) and eUSS positions (1-17) on the horizontal axis as sequence logo with the 9-mer USS underlined. Φ correlations according to given scale (0 to 1), with pairwise positions without significant correlations in blue.

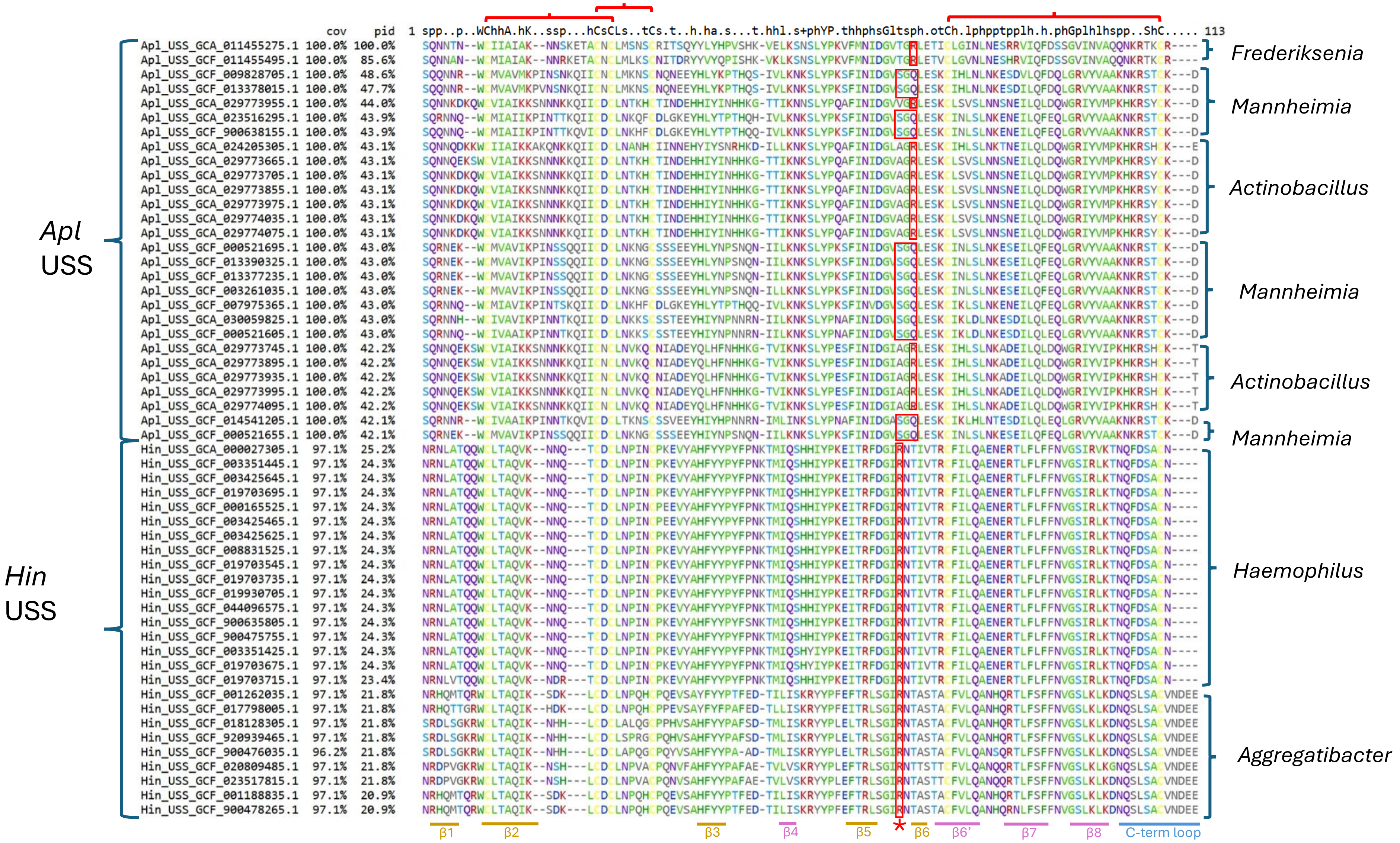

Figure S9. Partial $PpdA_{Past}$ alignment showing conserved and variable amino acids in the globular domain across five Pasteurellaceae genera highly enriched in USS. The structural protein domains are assigned. Disulfide bridges are marked with red connectors and an Arg147 of the Hin-USS clade located in the in the β5β6-loop is boxed and marked with *.

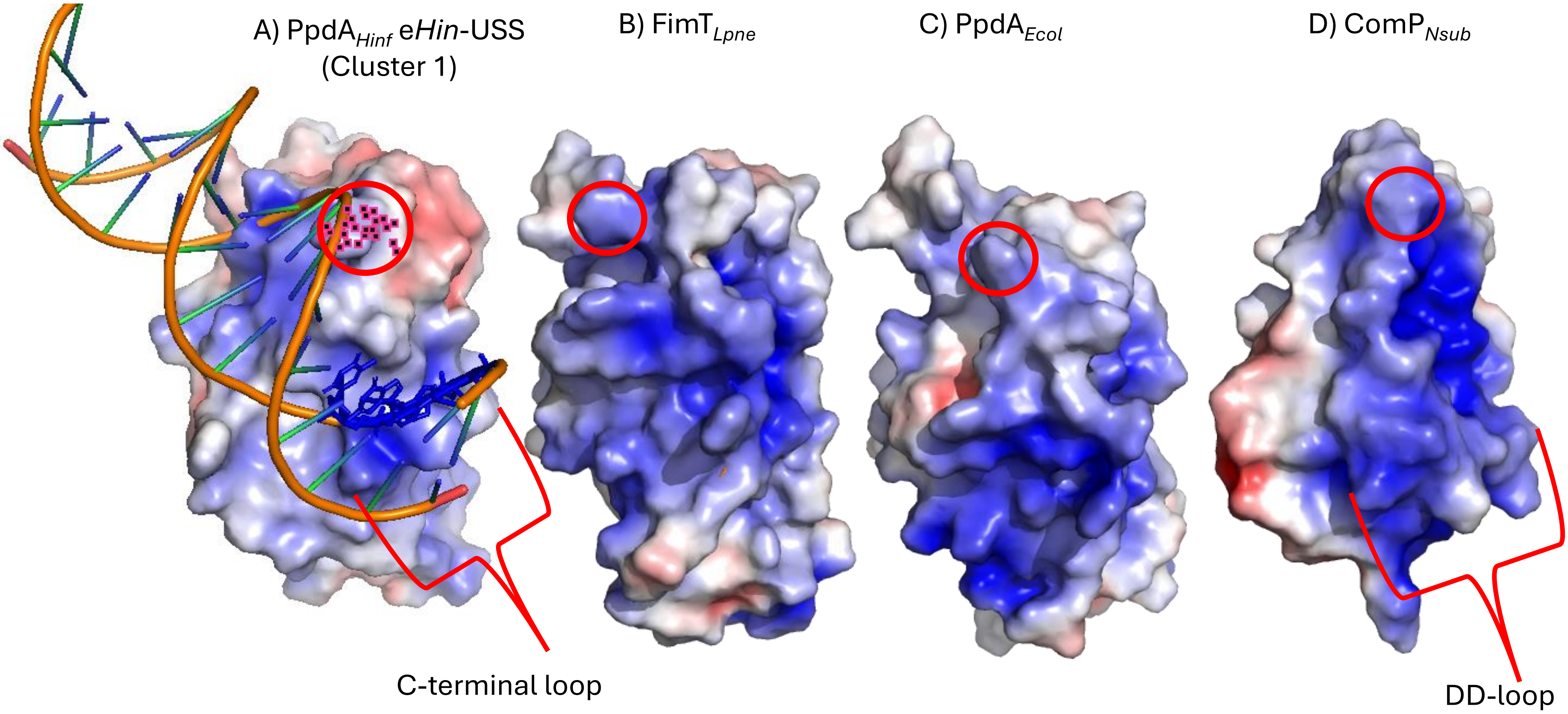

Figure S10. Electrostatic maps of established and candidate DNA binding minor pilins PpdA$_{Past}$-eUSS, FimT$_{Lpne}$, PpdA$_{Ecol}$ and ComP$_{Neis}$. A special positively charged Arg/Lys residue (Arg147 in the *Hin*-USS clade) is highlighted in the four structures proposed a unifying feature in unspecific DNA binding. The C-terminal loop of PpdA$_{Past}$ proposed involved in DNA binding specificity is marked in red brackets together with the DD-loop of ComP previously shown involved in sequence-specific DNA binding.

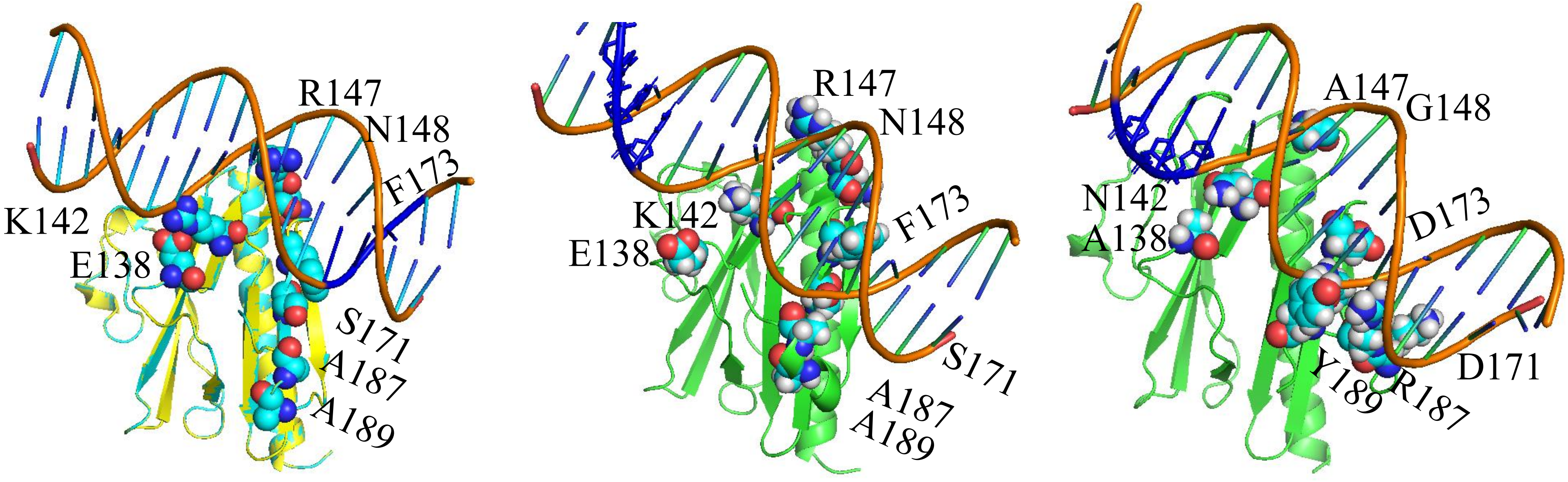

PpdA$_{eHinf\text{-}USS}$
H. seminalis SZY-H68

PpdA$_{eHinf\text{-}USS}$
H. parainfluenzae M1C130

PpdA$_{eApl\text{-}USS}$
A. equuli 3524

Figure S11. Strongly coevolved positions modeled in proximity of eUSS in two opposite orientations.
The eUSS-dialect variable positions 3-5 are shown in blue on the DNA which are modeled to reside in two alternative positions in cluster 1 (left) and cluster 2 (two right) of AF3 PpdA$_{\mathrm{eUSS}}$ complexes. Amino acid numbers refer to the PpdA alignment positions and the corresponding amino acids are labelled. In the PpdA$_{eHinf\text{-}USS}$ *H. seminalis* SZY-H68 of cluster 1 the eUSS dialect variable positions are modelled to interact with residues in the C-terminal β-sheet region involving coevolved F173 with DNA intercalating properties. In PpdA$_{eHinf\text{-}USS}$ *H. parainfluenzae* M1C130 and PpdA$_{eApl\text{-}USS}$ *A. equuli 3524* of cluster 2, the eUSS dialect variable positions are modelled to interact towards the N-terminal β-sheet region involving Asn138 with specific DNA binding properties.

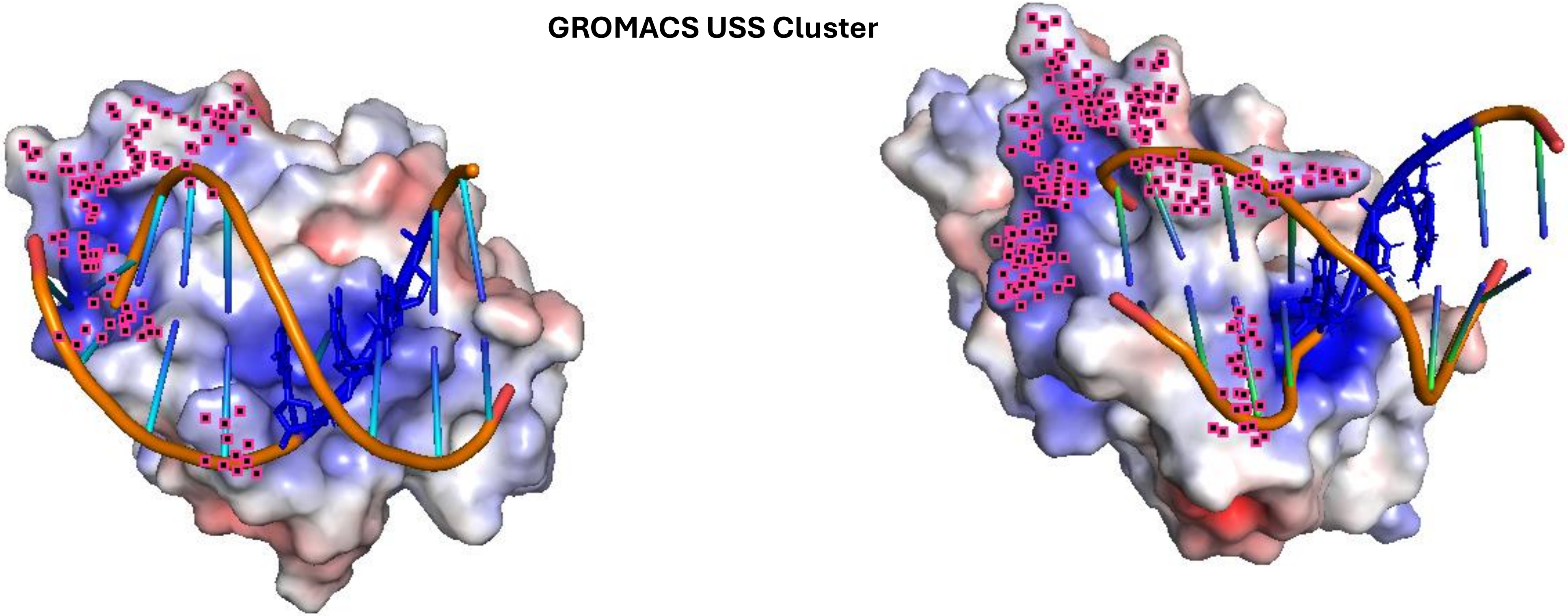

Figure S12. Molecular dynamics simulation of the interaction between PpdA$_{Hpar}$ (*Haemophilus parainfluenzae*) and9-mer *Hin*-USS as best confidence scored representative for the largest cluster of PpdA$_{USS}$ AF3 models. The input structure is shown to the left and the resulting structure to the right. The penultimate 9 C-terminal amino acids and R131 (R147 in PpdA alignment) are highlighted in pink. *Hin*-USS specific nucleotides are shown in blue. The input structure is the short 9-mer USS equivalent to Cluster 1 of the eUSS models and the simulation has brought the USS-dialect specific nucleotides more tightly into the electropositive pocket over the C-terminal β-sheet.

GROMACS Cluster 2

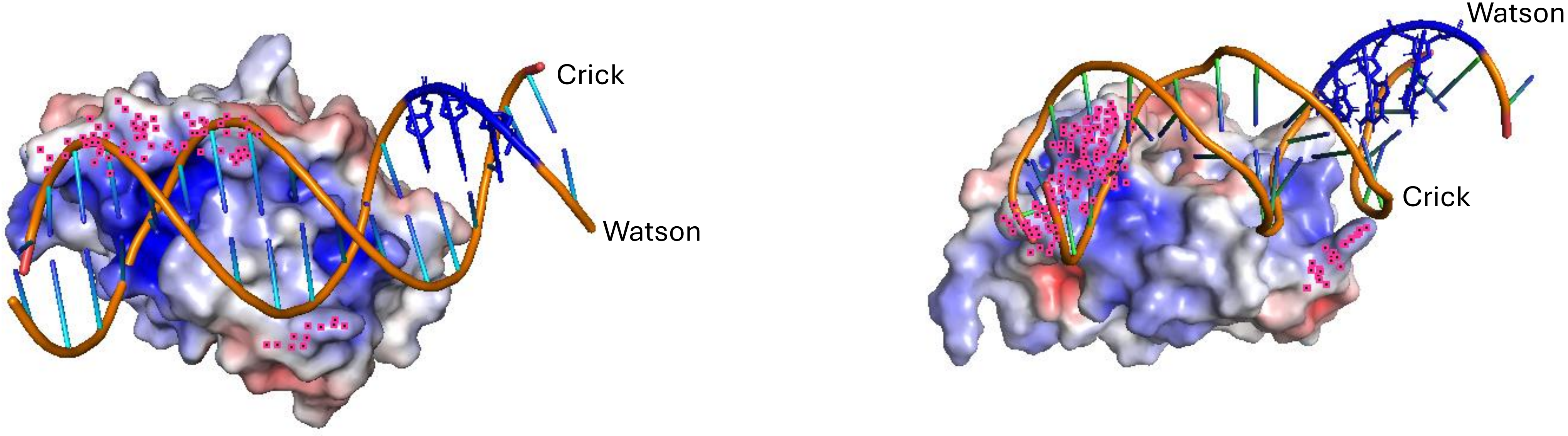

Figure S13. Molecular dynamics simulation of the interaction between PpdA$_{Hpar}$ (*Haemophilus parainfluenzae*) and 17-mer *Hin*-eUSS as best confidence scored representative for the largest cluster of PpdA$_{eUSS}$ AF3 models. The input structure is shown to the left and the resulting structure to the right. The penultimate 9 C-terminal amino acids and R131 are highlighted in pink. Hin-USS specific nucleotides are shown in blue. Both protein and DNA shapes have changed during the molecular simulation and seems to have narrowed the minor groove and widened the major groove accordingly. The USS-dialect specific nucleotides remains at some distance to the protein relative to cluster 1 and the less conserved AT-rich region beyond the 9-mer core USS is the DNA region which is the most integrated part of the structure .

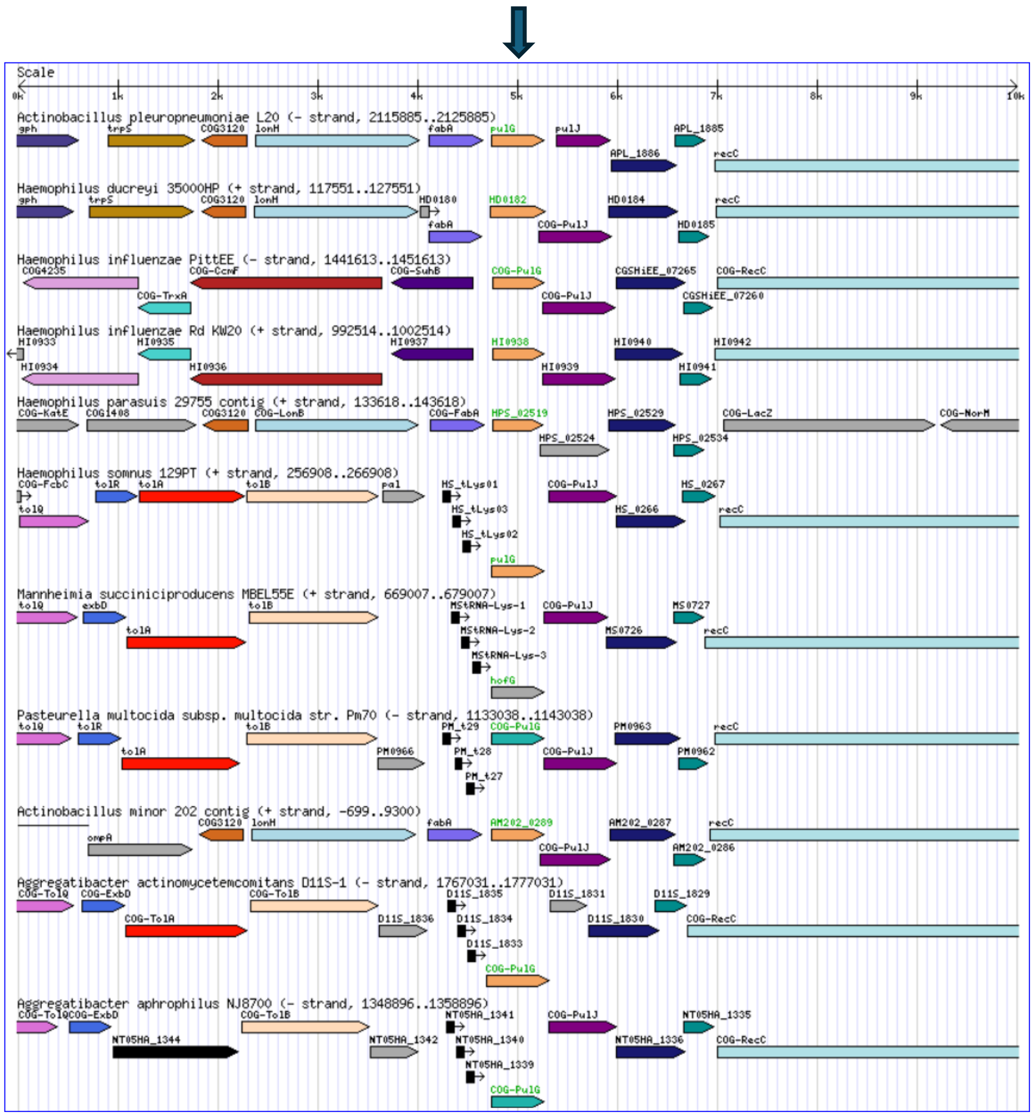

Image source: microbesonline.org

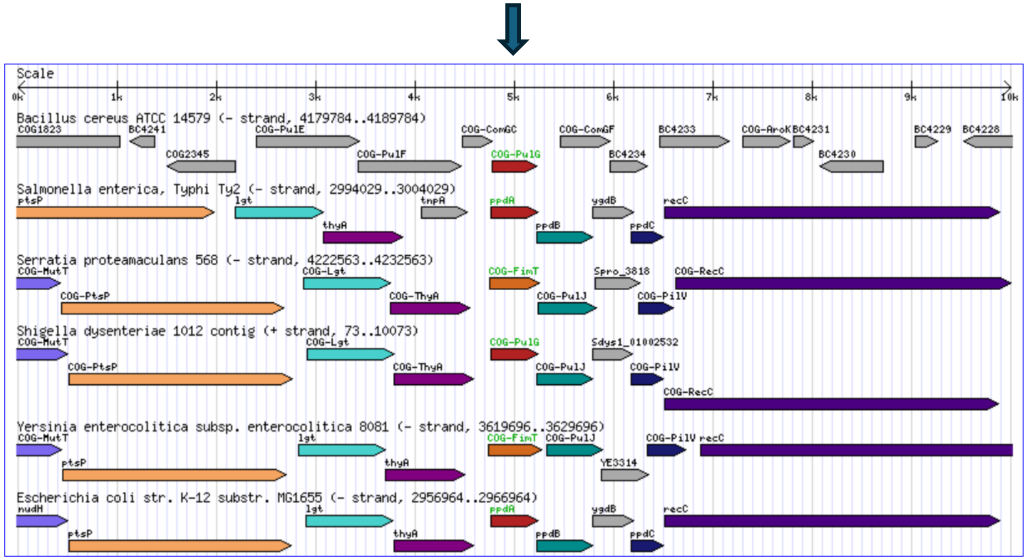

Figure S14. The genomic location and operonic organization of the *ppdA* gene across selected Pasteurellaceae family genera (left panel) and Enterobacteriaceae family genera (right panel) and the differently organized Gram positive *Bacillus cereus*. *ppdA* gene orthologs are marked with arrows as the first gene in the *comNOPQ* operon. The *ppdA* genes are annotated under different names including *pulG* and *fimT.* Images were generated by MicrobesOnline.org as described in (2).

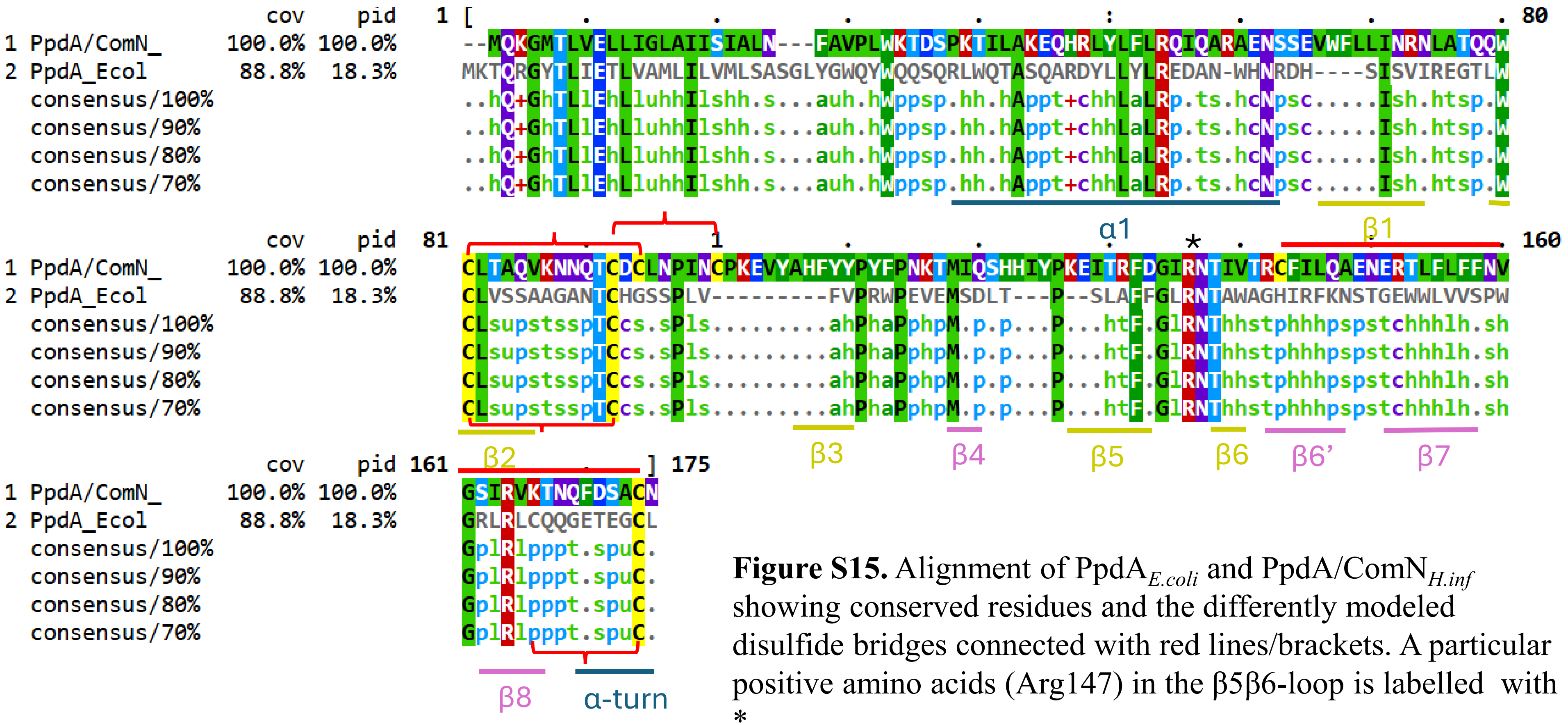

Figure S15. Alignment of PpdA$_{E.coli}$ and PpdA/ComN$_{H.inf}$ showing conserved residues and the differently modeled disulfide bridges connected with red lines/brackets. A particular positive amino acids (Arg147) in the β5β6-loop is labelled with *.
